# A Gq-Ca^2+^ Axis Controls Circuit-Level Encoding of Circadian Time in the Suprachiasmatic Nucleus

**DOI:** 10.1016/j.neuron.2013.03.011

**Published:** 2013-05-22

**Authors:** Marco Brancaccio, Elizabeth S. Maywood, Johanna E. Chesham, Andrew S.I. Loudon, Michael H. Hastings

**Affiliations:** 1 Division of Neurobiology, Medical Research Council Laboratory of Molecular Biology, Cambridge CB2 0QH, UK; 2 Faculty of Life Sciences, University of Manchester, M13 9PL, UK

## Abstract

The role of intracellular transcriptional/post-translational feedback loops (TTFL) within the circadian pacemaker of the suprachiasmatic nucleus (SCN) is well established. In contrast, contributions from G-coupled pathways and cytosolic rhythms to the intercellular control of SCN pacemaking are poorly understood. We therefore combined viral transduction of SCN slices with fluorescence/bioluminescence imaging to visualize GCaMP3-reported circadian oscillations of intracellular calcium [Ca^2+^]_i_ alongside activation of Ca^2+^/cAMP-responsive elements. We phase-mapped them to the TTFL, in time and SCN space, and demonstrated their dependence upon G-coupled vasoactive intestinal peptide (VIP) signaling. Pharmacogenetic manipulation revealed the individual contributions of Gq, Gs, and Gi to cytosolic and TTFL circadian rhythms. Importantly, activation of Gq-dependent (but not Gs or Gi) pathways in a minority of neurons reprogrammed [Ca^2+^]_i_ and TTFL rhythms across the entire SCN. This reprogramming was mediated by intrinsic VIPergic signaling, thus revealing a Gq/[Ca^2+^]_i_-VIP leitmotif and unanticipated plasticity within network encoding of SCN circadian time.

## Highlights

• SCN [Ca^2+^]i and TTFL circadian landscape phase-mapped by real-time imaging
• SCN network reprogrammed by recruitment of Gq-[Ca^2+^]i axis in a minority of neurons
• Selective Gq-mediated reprogramming mediated by intrinsic VIPergic signaling
• Internal structure and unanticipated plasticity of the SCN circadian network unveiled

## Teaser

Unraveling the circadian timekeeping properties of the suprachiasmatic nucleus has focused upon cell-autonomous transcriptional feedback loops. Brancaccio et al. now reveal that a Gq-[Ca^2+^]i axis encodes circadian time at the level of the suprachiasmatic neuronal circuitry.

## Introduction

Circadian rhythms pervade behavior and physiology, adapting organisms to the demands and opportunities of day and night, compromise of this temporal order having a major impact on health (Hastings et al., 2003; Takahashi et al., 2008). In mammals, circadian rhythms are coordinated by the hypothalamic suprachiasmatic nucleus (SCN), which sits atop a hierarchy of subordinate peripheral circadian clocks distributed across brain regions and organ systems. It is now well established that the SCN and peripheral clocks share a common circa 24 hr molecular timing mechanism, which is pivoted around transcriptional/posttranslational negative feedback loops (TTFL), in which the positive factors Clock and Bmal1 activate expression of the clock genes *Period (Per)* and *Cryptochrome (Cry)* via E box regulatory sequences. Subsequently, Per and Cry proteins suppress E-box activation, which can only recommence upon clearance of these negative regulators. Despite the success of the TTFL model in explaining circadian pacemaking within cells, an additional level of analysis is required to understand pacemaking across the SCN circuit, where interneuronal synchronization reinforces and augments the intracellular TTFL (Hastings et al., 2008; Mohawk and Takahashi, 2011). This augmentation is dependent upon intercellular neuropeptidergic cues (Liu et al., 2007; Maywood et al., 2011; Maywood et al., 2006), which in turn activate G protein-coupled receptors to regulate cytosolic signals, particularly cAMP- and calcium-dependent pathways (An et al., 2011; O’Neill et al., 2008). How these cytosolic signals relate to the TTFL and mediate their essential role in SCN pacemaking is poorly understood. Activation of Ca^2+^/cAMP-responsive elements (CREs), which is a point of convergence from upstream cytosolic pathways, in particular, cAMP and Ca^2+^, may play a role in this process (Bito et al., 1997). In the context of the SCN neuron, activation of CREs therefore provides a valuable report of the integrated afferent information received from the intercellular SCN network for transmission to the intracellular TTFL clockwork (Travnickova-Bendova et al., 2002).

To address the relationship between SCN circuitry, cytosolic signals, CREs, and the TTFL, we used viral transduction to deliver to organotypic SCN slices bioluminescence- and fluorescence-based reporters of cytosolic circadian rhythms. In this way we phase-mapped to the TTFL cytosolic rhythms of [Ca^2+^]_i_ and activation of CREs, in both circadian time and SCN space and found that they were abolished in circuits lacking VIP. Having characterized this program, and shown its dependence upon neuropeptidergic G-coupled signaling, we then sought to examine causal relationships within it. We took advantage of recent developments in pharmacogenetics (Rogan and Roth, 2011), by using DREADDs (designer receptor exclusively activated by designer drug) as a means to activate specific G-coupled pathways in subsets of SCN neurons. In contrast to optogenetic approaches, which have been especially useful for the control of neural activity in relatively short time-frames (Yizhar et al., 2011), DREADDs are eminently suited to manipulating neuronal function in the circadian time domain (Garner et al., 2012). Moreover, because organotypic SCN slices are a faithful representation in vitro of circadian time-keeping in vivo, they constitute a powerful model to explore the interplay between cell-intrinsic and circuit-based properties in the specification of a fundamental, adaptive behavior (Welsh et al., 2010). SCN somatic chimeras were therefore created by lentiviral (LV) transduction with constructs encoding DREADDs to activate Gq-, Gs-, or Gi-dependent signaling pathways in transduced cells (Armbruster et al., 2007; Rogan and Roth, 2011). We show that these pathways control the cytosolic and TTFL circadian components in particular and selective ways, affecting period, amplitude and coherence, not only at the level of individual cells but also across the SCN circuit. Remarkably, activating Gq-dependent pathways (but not Gs or Gi) in a minority of SCN neurons selectively reprogrammed the intracellular calcium and the downstream TTFL components propelling the circuit to a new defined state. Cocultures of SCN slices and an intersectional genetics approach that targeted DREADDs to VIP neurons demonstrated that this reorganization was mediated by the intrinsic, VIP subcircuit of the SCN network. In contrast to the existing view of the SCN as a resilient circadian pacemaker, the current study therefore reveals an unforeseen plasticity of its circuit-encoded properties through which it can operate in more than a single state. Thus, the Gq-Ca2^+^ axis is a key control point for network programming of SCN circadian time.

## Results

### Circadian Activation of Ca^2+^/cAMP-Responsive Elements in Wild-Type and Mutant SCN

**Figure 1:**
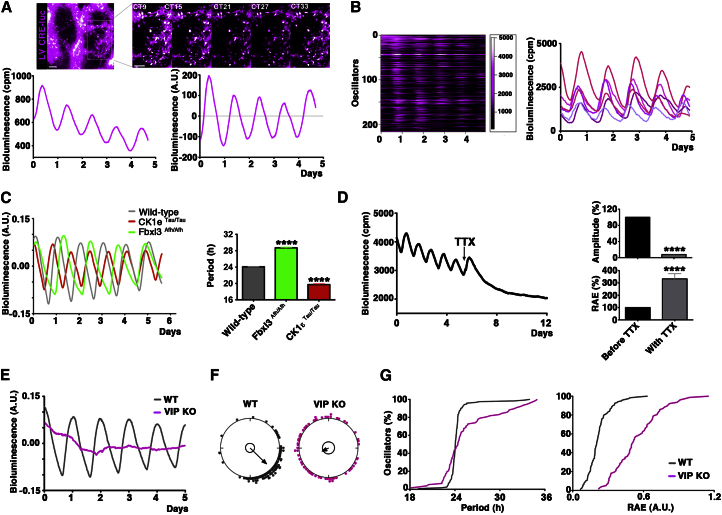
Circadian Activation of Ca^2+^/cAMP-Responsive Elements (CRE) in the SCN (A) Representative SCN slice transduced with LV:CRE-luciferase, showing numerous cells distributed across SCN that exhibit circadian oscillations (inset), reflected in the aggregate bioluminescence plot before (left) and after linear detrending (right). (B) Semiautomatic image analysis (SARFIA) reveals rhythmic CRE activation at single cell level, expressed as raster plot (left). CRE oscillations have “saw-tooth” waveform and slightly different phases across the slice (right). (C) Representative traces of PMT bioluminescence recording of WT, Fbxl3^Afh/Afh^ and CK1ε^Tau/Tau^ SCN transduced with the LV:CRE-luc reporter. All data mean + SEM, p < 0.0001, ANOVA with Bonferroni correction. (D) Representative trace of bioluminescence recording from SCN transduced with LV:CRE-luc and treated with TTX (0.5 μM) showing decreased amplitude and robustness (increased RAE values, right panel, mean ± SEM, p < 0.0001, n = 11, paired two-tailed t test). (E and F) De-trended traces and Rayleigh plots of representative WT and VIP^−/−^ SCN transduced with LV:CRE-luc, showing impaired overall rhythmicity and disrupted internal coupling of CRE oscillators (r mean vector CRE WT = 0.78, n = 120; VIP^−/−^ = 0.24, n = 72). (G) Cumulative plots reveal increase in period scattering and minimal robustness of the CRE rhythm (high RAE). Scale bars: 50 μm. See also Movie S1.

To monitor the activation of CREs across circadian time, SCN slices were transduced with LVs encoding a firefly-luciferase reporter controlled by a minimal synthetic promoter containing two CREs (LV:CRE-luc) and deprived of any other regulatory sequences. CCD real-time imaging identified significant numbers of transduced cells (mean ± SEM = 128 ± 26 cells/SCN n = 5; Figures 1A and 1B; see Movie S1 available online). These cells were sufficiently scattered across the SCN to facilitate semiautomated image analysis (SARFIA), which revealed pronounced circadian rhythmicity, synchronized across the slice. Interestingly, the activation of CRE displayed a distinctive “saw-tooth” asymmetric waveform (nadir-zenith = 10.25 ± 0.25 hr; zenith-nadir = 14.00 ± 0.29 hr, n = 5; Figure 1B), which contrasts with the typically sinusoidal pattern of TTFL reporters. The period of the CRE rhythm was significantly accelerated by the period-shortening CK1ε^*Tau*^ mutation, and lengthened by the Fbxl3^*Afh*^ mutation (Figure 1C), demonstrating the role of the TTFL in setting the pace of cytosolic signaling rhythms reported by activation of CRE.

Treatment with the sodium channel blocker tetrodotoxin (TTX) to stop action-potential firing and thereby silence interneuronal signaling progressively decouples and damps the circadian TTFL rhythms in SCN cells (Yamaguchi et al., 2003; Hastings et al., 2007). Consistent with a role for CRE elements in integrating circuit-level stimuli and transducing their action onto the TTFL, TTX treatment immediately suppressed and disorganized circadian rhythms of CRE activation (Figure 1D). Such circuit-level signaling is heavily reliant upon the neuropeptide VIP, which is essential for neuronal coupling within the SCN (Atkinson et al., 2011; Aton et al., 2005; Harmar et al., 2002; Maywood et al., 2011; Maywood et al., 2006). VIP acts via VPAC2 receptors, which in turn regulate Gs-cAMP and Gq-Ca^2+^ pathways (An et al., 2011; Dickson and Finlayson, 2009). Thus, VIP/VPAC-mediated interneuronal signals may act via CRE activation. Consistent with this, SCN slices lacking VIP showed dramatic impairments in CRE circadian rhythms (Figure 1E), comparable to those seen under TTX. When compared to WT, VIP^−/−^ SCN exhibited fewer detectably rhythmic cells (31% versus 83%). Moreover, rhythmic cells showed a much higher degree of phase scattering, evidenced by Rayleigh plots (Figure 1F). In the uncoupled VIP -null circuit, the circadian period of detectable CRE oscillators was highly dispersed and the robustness of their oscillation dramatically impaired (Figure 1G). Thus, the absence of circuit-level electrical activity or VIPergic signaling impaired the circadian oscillations of CREs, consistent with their role in conveying circuit-wide stimuli to the intracellular TTFL, and thereby reinforcing SCN circadian rhythmicity.

### Selective Roles for Gs, Gi, and Gq Signaling Pathways in Controlling Circadian Rhythms of CRE Activation in SCN

To determine the relative contributions of different G-coupled pathways in mediating circuit-wide signals, we used a pharmacogenetic approach to stimulate, individually, Gq, Gs, and Gi in SCN neurons. Three different LVs encoding a DREADD receptor (Rogan and Roth, 2011) designed to activate Gq, Gs, or Gi signaling (respectively LV: Syn-hM3DGq-IRESmCherry, LV:Syn-rM3/β1Gs-IRESmCherry, LV:Syn-hM4DGi-IRESmCherry) were used to transduce SCN slices. LV:CRE-luc was cotransduced, in order to report both the acute and tonic changes following stimulation of each pathway.

**Figure 2:**
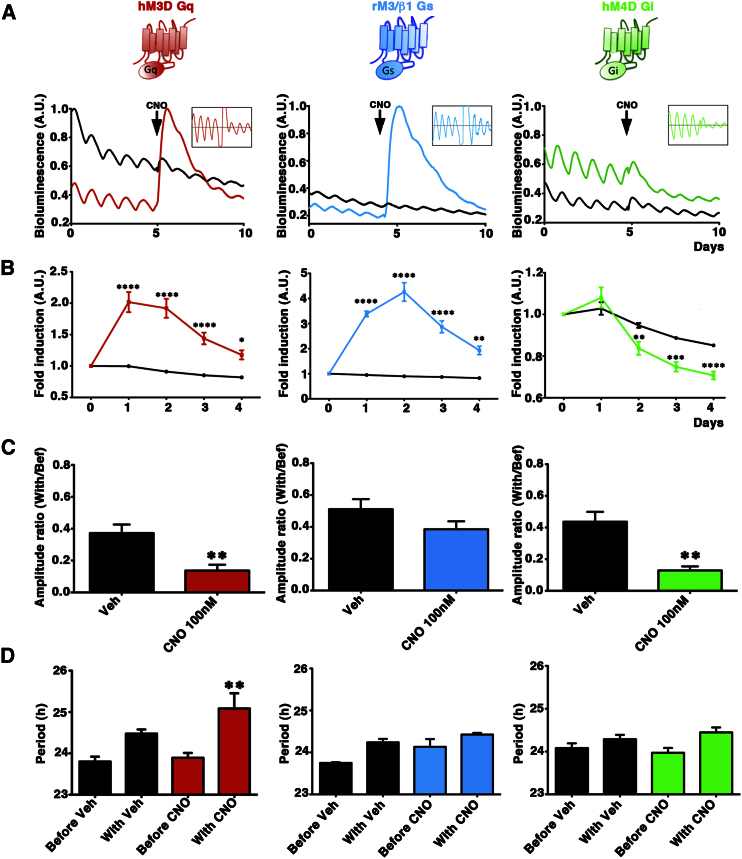
Selective Roles for Gs, Gi, and Gq Signaling Pathways in Controlling Circadian Rhythms of CRE Activation in SCN (A) Representative bioluminescence recordings from SCN slices transduced with the LV:CRE-luc reporter and LV-DREADDs: Syn-hM3DGq-IRESmCherry, LV:Syn-rM3/β1Gs-IRESmCherry or LV:Syn-hM4DGi-IRESmCherry, respectively. CNO (100 nM) triggered Gq- and Gs-stimulated increases or a Gi-stimulated decrease of CRE activation. Insets show detrended traces to reveal the circadian oscillations before and in the presence of CNO. (B) Relative induction/suppression of CRE activation plotted by day of treatment (mean ± SEM, n = 3–6 per group). For all CNO/DREADD treatments, two-way ANOVA, p < 0.5 = ^*^; p < 0.01 = ^**^; p < 0.001 = ^***^p < 0.001 = ^****^ versus initial activity. (C) Gq and Gi activation decreased the amplitude of the CRE rhythm, whereas no significant changes are observed following Gs stimulation (mean + SEM, two-tailed t test). (D) Activation of Gq pathway significantly lengthened period of CRE-luc rhythm (p < 0.01 n = 6). Stimulation of Gs or Gi signaling had no significant effect on period (mean + SEM, n = 3–6 per group, two-way ANOVA, ^**^ = p < 0.01 versus other measures). See also Figure S1.

In the absence of their specific ligand, Clozapine-N-Oxide (CNO), DREADD receptors are silent and accordingly, CRE activity exhibited unperturbed circadian cycles (Figure 2A; note compressed ordinate scale) and vehicle addition had no effect on CRE circadian activation. Stimulation of the DREADDs with CNO, however, had marked and selective effects (Figures 2A and 2B). Consistent with the cAMP-mediated control of CRE, induction of Gs (activating adenylate cyclase) and Gi (inhibiting adenylate cyclase) significantly increased and suppressed CRE activity, respectively. Activation of Gq-dependent pathways also stimulated CRE, although to a lesser degree than Gs (Figure 2B). Sustained DREADD stimulations had further selective effects on the SCN circadian clock. Whereas both Gq and Gi activation significantly reduced the amplitude of the CRE oscillations, Gs activation did not (Figure 2C). Remarkably, Gq activation specifically lengthened the period by ∼1.2 hr, an effect absent in Gs or Gi activated slices (Figure 2D). These data suggest a deeper effect on the SCN circadian clock selectively exerted by activation of Gq signaling. Importantly, CRE induction and period lengthening by Gq stimulation were dose dependent (Figures S1A–S1C). Treatment with CNO (100 nM) for 1 hr was sufficient to decrease the amplitude of the CRE rhythm and 24 hr treatment lengthened period (Figures S1E and S1F). Finally, acute CRE-luc induction fell on washout, indicating effective CNO removal. These results confirm the specificity of the respective DREADDs and highlight the central position of CRE activation as a convergence point of G-coupled cytosolic signaling cascades in the SCN. Moreover, by revealing distinct and separable contributions of Gs, Gi, and Gq within the circadian pacemaker they also highlighted a pivotal contribution of Gq pathways to circadian properties of CRE activation.

### Gq Activation Reorganizes Circadian *Per1* Expression through CRE Recruitment within the SCN Circuit

**Figure 3:**
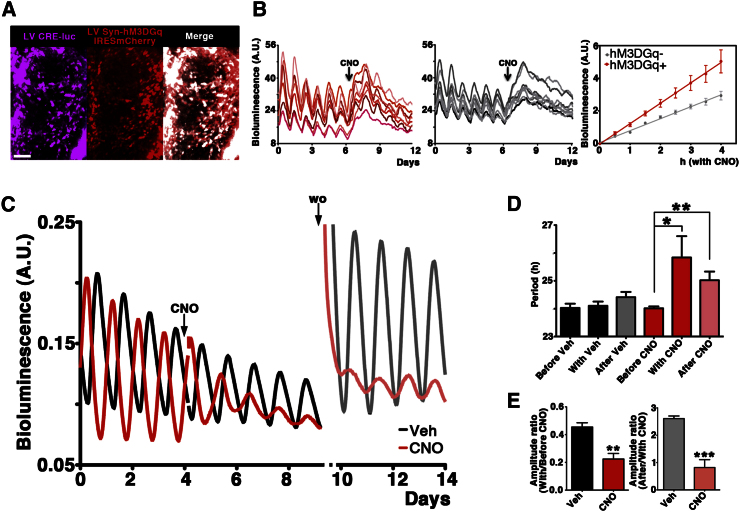
Gq Activation Reorganizes Circadian CRE Activation and *Per1* Expression across the Entire SCN Circuit (A) Representative SCN transduced with LV:CRE-luc and Syn-hM3DGq-IRESmCherry: merge reveals single and double transduced cells. (B) CNO-stimulated CRE activation was present both in Syn-hM3DGq^+^-cells (red, left) and the hM3DGq^−^ population (gray, middle). Note significantly slower kinetics of induction in DREADD-negative cells (right). (C) Representative traces from *Per1*-luc SCN transduced with the LV:Syn-hM3DGq-IRESmCherry and treated with either vehicle (black) or CNO (red) (100 nM, CNO) followed by washout (wo) with fresh medium after 5 days. (D) Mean data (+SEM) of circadian period before, during and after treatment with vehicle (black/gray) or CNO (red). Note significant lengthening with CNO (p < 0.05 n = 4, two-way ANOVA with Bonferroni correction), but not vehicle (n = 5) that is sustained after washout (p < 0.01). (E) Mean data (+SEM) of relative amplitude of circadian CRE activation revealed ca. 50% reduction both during CNO (left, p < 0.01 two-tailed t test) and after CNO removal (right, p < 0.001). Scale bar = 50 μm.

As previously shown (Figure 1A), although LV transduced cells in our chimeric slices are a minority (∼150 cells), nevertheless, the effects exerted by DREADD-mediated Gq stimulation consistently and dramatically altered the overall CRE rhythm recorded by PMT, suggesting that circuit-wide effects may account for the phenotype observed. To test this, SCN transduced with LV:CRE-luc and LV:Syn-hM3DGq-IRESmCherry were followed on camera by multichannel real-time imaging (Figure 3A), thereby allowing cellular CRE rhythms to be assigned to DREADD-positive or DREADD-negative subpopulations. Before addition of CNO, all analyzed cells exhibited clear circadian cycles of CRE activation (Figure 3B). As anticipated, CNO directly activated CRE across the SCN in cells expressing the DREADD. However, it also activated CRE in cells without detectable fluorescence, thus revealing an indirect effect. Significantly, this occurred with slower kinetics, indicative of a trans-neuronal delay (rates of bioluminescence increase, A.U./hr: DREADD^+^ cells = 0.62 ± 0.04, DREADD^−^ cells = 0.37 ± 0.02, p < 0.01, n ≥ 8; Figure 3B). Thus, Gq activation in cells expressing the DREADD receptors recruited CRE activation in other, DREADD-negative oscillators, affecting circadian behavior of both transduced and nontransduced cells.

Having revealed the effects of Gq stimulation on the circadian dynamics of CRE activity, we then tested if the effects of Gq upon SCN circadian behavior were propagated to TTFL components. The effects of DREADD-mediated Gq activation were therefore tested on SCN transgenic for *Per1*-luc. This reporter carries several CREs and also several E-boxes (Yamaguchi et al., 2003), and would thus report both the acute effects of Gq activation and longer term, E-box-dependent changes within the TTFL (Travnickova-Bendova et al., 2002).

In contrast to vehicle, CNO activation of Gq increased baseline *Per1*-luc bioluminescence in the subsequent cycle, and this elevation was sustained until washout, consistent with the response of CRE-luc noted above (Figure 3C). Moreover, the amplitude of the rhythm was reduced and the period significantly lengthened (Figures 3D and 3E). Thus, the circadian effects of Gq activation upon CRE were extended to *Per1*-luc, indicating that Gq-dependent signals gain access to the E-box-dependent core transcriptional feedback loops. Surprisingly, CNO removal did not restore the period or amplitude of the *Per1*-luc rhythm: the circadian consequences of Gq activation were not reversible.

The germ-line-encoded *Per1*-luc transgene is present in all cells and we could define 480 ± 10 cells per SCN (mean ± SEM, n = 3) actively expressing it. In the same slices 186 ± 33 cells were somatically transduced by the LV:Gq vectors with a maximal theoretical transduction rate of 39%. This is, however, to be considered an overestimate—first, because only a fraction of the cells in the slice express *Per1*-luc (Yamaguchi et al., 2003) and, second, the difficulty of resolving the individual bioluminescent signals from densely packed cells undercounts them. We therefore anticipate a real transduction rate of <20%. Thus, the phenotype could not be ascribed to the DREADD-transduced subpopulation alone. Rather, Gq permanently reorganized the circadian program across the chimeric SCN. Taken together, these data demonstrate that interneuronal signals, activated by Gq/CRE signaling in transduced cells, can modify the circadian properties of downstream neurons, extensively changing the behavior of the whole SCN circuit.

### Phase-Mapping Intracellular Calcium to the TTFL Unveils the SCN Circadian Program

Gq-mediated induction of CRE was significantly less than that following Gs activation (Figure 2B), but nevertheless it respecified circadian parameters, suggesting that acute CRE induction per se is not the mechanism behind the Gq effect. Consistent with this, treatment with forskolin/IBMX also activated CREs but did not affect circadian period or amplitude (Figures S2A and S2B). The Gs/forskolin pathway is specifically linked to cAMP, whereas Gq signaling is linked to intracellular calcium ([Ca^2+^]_i_), via phospholipase C. We therefore hypothesized that the circadian consequences of Gq stimulation may arise from its specific effects on upstream cytosolic signals, in particular [Ca^2+^]_i_.

**Figure 4:**
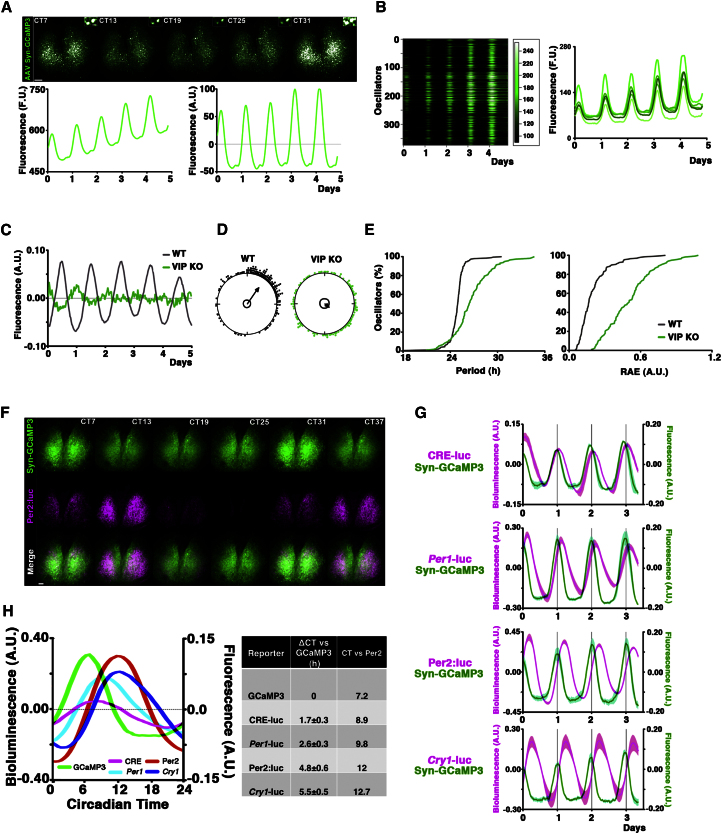
Circadian Rhythm of Intracellular Calcium in SCN, Phase-Mapped to the Circadian TTFL (A) Serial images of SCN slice transduced by AAV-Syn-GCaMP3 showing high rates of neuronal transduction and high GCaMP3 expression levels (inset: single cells in the slice). Below is aggregate circadian rhythm of [Ca^2+^]_i_ before (left) and after (right) de-trending (period = 23.81 ± 0.12 hr, mean ± SEM, n = 14). (B) SARFIA analysis showing rhythms in single cells plotted as raster (left) and graphical (right) plots. Note that the distinctive waveform of the GCaMP3^+^ aggregate trace is evident in individual neurons. (C) Detrended traces and Rayleigh plots of representative WT and VIP^−/−^ SCN transduced with AAV:Syn-GCaMP3, showing similar results to the CRE (r mean vector GCaMP3: WT = 0.66 n = 191, VIP^−/−^ = 0.18, n = 112). (E) Cumulative plots of GCaMP3 oscillations displaying increase in period scattering and minimal robustness of the rhythm (high RAE) in VIP^−/−^ SCN. (F) Serial images from Per2:luc knock-in SCN transduced with AAV-Syn-GCaMP3, showing different peaking times. (G) Circadian rhythms of bioluminescence and fluorescence from SCN transduced with AAV:Syn-GCaMP3 and expressing CRE-luc, *Per1*-luc, Per2:luc or *Cry1*-luc reporters, respectively. Data plotted as mean ± SEM (n ≥ 3) for each configuration. Interpeaks distance (ΔCT versus GCaMP3) is used to align the various rhythms around the circadian day. (H) Phase map of circadian cytosolic oscillations and TTFL based on normalized cycles for each component. Scale bars: 50 μm. Inset: 10 μm. See also Movies S2 and S3.

To directly identify neuronal [Ca^2+^]_i_ rhythms, SCN slices were transduced with adenoassociated vectors (AAVs) encoding the fluorescent reporter GCaMP3 controlled by the neuronally restricted synapsin promoter (AAV:Syn-GCaMP3) (Tian et al., 2009). One week after transduction, strong signal was evident in neurons across the SCN and overall fluorescence was highly circadian, with estimated [Ca^2+^]_i_ ranging between 113 ± 7.5nM (nadir) and 191 ± 25nM (zenith, mean ± SEM n = 3, see Experimental Procedures; Figure 4A; Movie S2). The circadian oscillation of [Ca^2+^]_i_ progressed with a spatiotemporal wave, initiated dorsomedially adjacent to the third ventricle, and passing ventrolaterally. Its circadian waveform, however, was distinct from that of both CRE and TTFL reporters, with a prolonged trough and relatively brief peak (respectively 15.5 ± 0.2 hr versus 8.4 ± 0.4 hr, n = 5; Figure 4A; Movie S2). As with the saw-tooth activation of CREs, this distinctive waveform was reproduced at the level of individual neurons analyzed by SARFIA, showing a range of different phases accumulated around the overall average slice peak (Figures 4B and 4D). Consistent with a potential role for [Ca^2+^]_i_ rhythms as mediators of interneuronal signals, [Ca^2+^]_i_ circadian rhythms were dramatically impaired in VIP null SCN (Figures 4C–4E). At the slice level the pattern was extremely damped and overall fewer rhythmic cells were detected (VIP null 61% versus WT 89%). Moreover, the phases of the detectably oscillating cells were highly dispersed, as evidenced by Rayleigh plots (Figure 4D). The period of individual cellular [Ca^2+^]_i_ rhythms within the uncoupled slice was also highly dispersed and the robustness of the oscillations dramatically impaired (Figure 4E). Thus, the circadian control of [Ca^2+^]_i_ rhythms faithfully recapitulated the phenotype of the downstream rhythms of CRE activation (Figures 1F and 1G).

To define more clearly the temporal relationships between circadian rhythms of [Ca^2+^]_i_ and CRE, and place them in the wider context of the TTFL, we phase-mapped these events in SCN space and circadian time. Combined bioluminescence and fluorescence imaging (Figures 4F–4H) was used to define the mutual phase relationship between [Ca^2+^]_i_, CREs and a selection of previously characterized, genetically encoded bioluminescent TTFL reporters (*Per1*-luc [Yamaguchi et al., 2003], Per2:luc [Yoo et al., 2004], *Cry1*-luc [Fustin et al., 2009]; Figure 4G; Movie S3). These relationships were stable, and cross-registration to the GCaMP3 peaks made it possible to assemble all of the bioluminescently reported events into a single, comprehensive phase-map of the SCN circadian program. In addition, because Per2 expression peaks at circadian time (CT) 12 (Field et al., 2000), we used the expression of the Per2:luc fusion-protein reporter to assign absolute CT values to the cytosolic and TTFL events (Figure 4H).

This phase-mapping showed that [Ca^2+^]_i_ peaked around CT07, anticipating the CRE-luc peak by 1.7 ± 0.3 hr (all reporters, mean ± SEM, n ≥ 3). This was followed by *Per1*-luc (2.6 ± 0.3 hr), which carries highly effective CREs (Travnickova-Bendova et al., 2002) (plus E boxes), the Per2:luc posttranslational reporter (4.8 ± 0.6 hr), with (less effective) CREs (and E boxes), and finally the *Cry1*-luc (5.5 ± 0.5 hr), which is devoid of CREs but carries E boxes (Fustin et al., 2009; Figure 4H). Given that GCaMP3 reports [Ca^2+^]_i_ instantaneously but bioluminescent reporters incorporate a lag for transcription and translation of the enzyme (ca. < 1 hr), the phase-map indicates that the circadian peak of [Ca^2+^]_i_, at CT07 is followed soon afterward by CRE activation (ca. CT08) and subsequently by the CRE-containing TTFL components, *Per1* and then Per2. *Cry1*-luc, lacking CREs, became active significantly later (ca. 4 hr after CRE-luc). Thus, a precisely timed SCN circadian program can be outlined, starting with an abrupt arise of [Ca^2+^]_i_ that precedes an acute activation of CRE followed by more sinusoidal changes in TTFL components.

### Gq Signaling Selectively Reprograms [Ca^2+^]_i_ across the SCN Circuit

**Figure 5:**
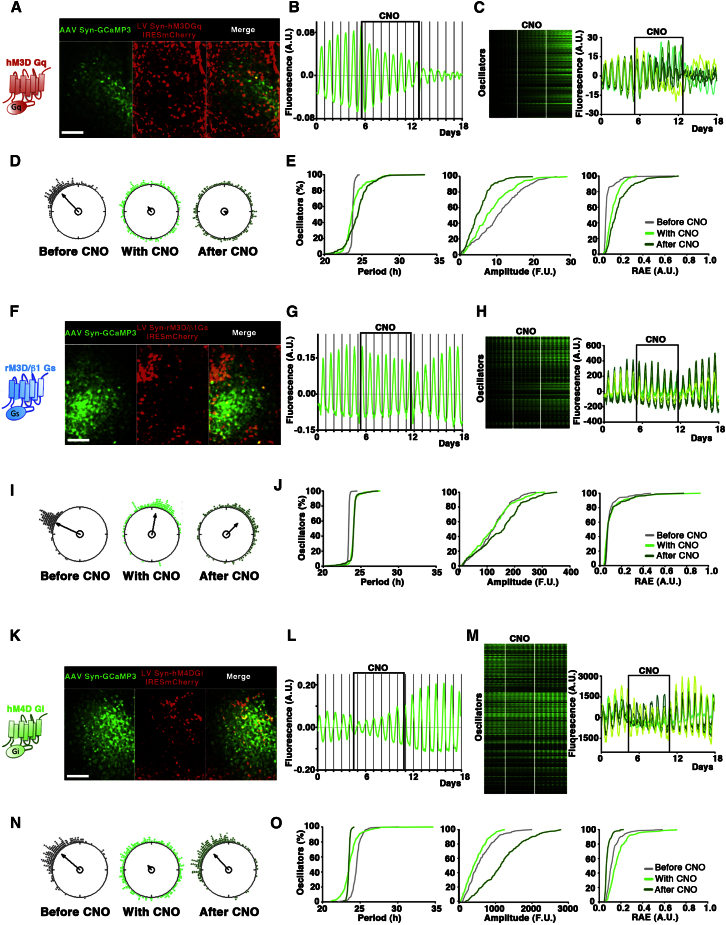
Selective Reprogramming of [Ca^2+^]_i_ Circadian Rhythms by Gq Signaling across the SCN Circuit (A, F, and K) Representative SCN slices transduced with AAV:Syn-GCaMP3 and LV:Syn-hM3DGq-IRESmCherry, LV:Syn-rM3/β1Gs-IRESmCherry or hM4DGi-IRESmCherry, respectively. Microphotographs showed that only a subset of the GCaMP3^+^ neurons also expressed the DREADD receptors. (B) Detrended trace of the SCN aggregate fluorescence signals from (A) before, during and after CNO treatment, shows irreversible reduction of amplitude of GCaMP3 oscillations. (C) Raster plots and representative traces of GCaMP3 from individual cells in (A) showing both loosened overall synchrony and reduced amplitude and robustness in single cells. (D) Rayleigh plots confirm desynchronization of SCN cells within the slice induced by Gq, an effect not reversed by CNO removal. (E) Cumulative plots showing that Gq activation irreversibly increases period and decreases the amplitude and coherence (RAE) of individual cellular rhythms. (G) Detrended trace of the aggregate fluorescence signals from the culture in (F) shows that activation of Gs signaling did not alter significantly overall GCaMP3-reported [Ca^2+^]_i_ rhythms. (H) Raster plots and representative traces of cellular GCaMP3 recordings show that Gs stimulation did not affect cellular GCaMP3 fluorescence rhythms. (I) Rayleigh plots reveal slight reduction of the phase coupling of cellular [Ca^2+^]_i_ rhythms, elicited by Gs stimulation. (J) Cumulative plots demonstrate that stimulating Gs has little effect on cellular period, amplitude, or coherence (RAE). (L) Detrended trace of the aggregate fluorescence signals from the SCN in (K) in the presence of CNO and after drug removal. Stimulation of Gi transiently diminished the overall GCaMP3 rhythm amplitude, but CNO removal reversed the phenotype, with rhythmic amplitude exceeding the pretreatment values. (M) Raster plots and representative traces of rhythms show that Gi reduced [Ca^2+^]_i_ amplitude in single cells, but this effect was reversed on CNO removal. (N) Rayleigh plots displaying transient reduction of the phase coupling of GCaMP3^+^ oscillators by Gi, reversed by CNO washout. (O) Cumulative plots showing transiently decreased amplitude and increased RAE of the [Ca^2+^]_i_ cellular rhythms in the presence of CNO, returned to, or even exceeded original pretreatment levels, after drug removal. Scale bars: 50 μm. See also Table S1, Figure S2, and Movies S4, S5, and S6.

Having shown that circadian oscillations of [Ca^2+^]_i_ in the SCN anticipated peak activation of CREs and so might mediate the reprogramming effects of G-coupled signals upon CRE, we examined the effects of the individual DREADDs upon intracellular calcium rhythms. SCN slices were transduced with LV:Syn-hM3DGq-IRESmCherry, LV:Syn-rM3/β1Gs-IRESmCherry, LV:Syn-hM4DGi-IRESmCherry and the effects of stimulation of each G pathway on [Ca^2+^]_i_ were followed with the GCaMP3 reporter (Figure 5). Gq activation immediately and progressively reduced the amplitude of the overall oscillation (Figures 5A and 5B). This reduction was a consequence of dramatic desynchronization between the oscillators, as well as decreased amplitude and robustness of the rhythm in single cells. Period dispersal of the rhythm within the population was increased and shifted toward longer periods. None of these effects were reversed by washout of CNO, thus confirming the irreversible reprogramming of the SCN circuit (Figures 5C–5E; Movie S4; Table S1). In contrast, stimulation of Gs did not alter significantly the overall GCaMP3 rhythm. At the cellular level no significant changes of the calcium oscillations were detectable, and no reduction in amplitude or robustness. Period dispersal was equally unaffected, and phase scattering only slightly increased (Figures 5F–5J; Movie S5; Table S1). Finally, Gi activation transiently reduced the amplitude, phase synchrony and robustness of the [Ca^2+^]_i_ rhythm, but upon CNO removal the cellular rhythms reverted to pre-treatment patterns (Figures 5 K–5O; Movie S6; Table S1). Interestingly, after CNO removal the amplitude and the robustness of GCaMP3 oscillators were increased when compared to pretreatment values, suggesting reinforcement of the intracellular [Ca^2+^]_i_ oscillations upon drug washout (Figure 5O). Thus, although Gs can acutely activate CRE more than Gq signaling, it does not significantly alter intracellular calcium, whereas Gq stimulation elicited a major reorganization of [Ca^2+^]_i_, both in cells and across the circuit.

### Gq activation Reprograms the Global Spatiotemporal Dynamics of the SCN TTFL to a New Specific State

**Figure 6:**
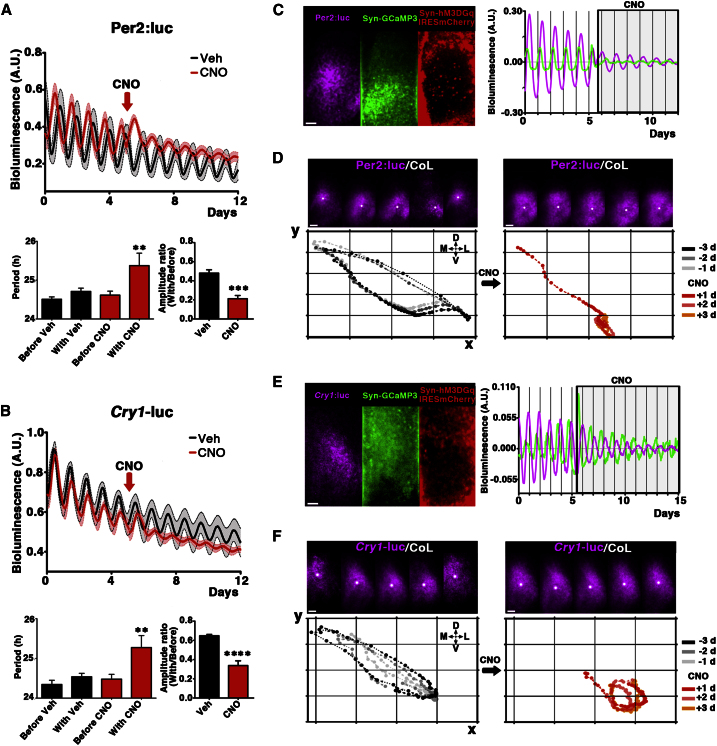
Gq Activation Reprograms the Global Spatiotemporal Dynamics of the SCN TTFL (A and B) Recordings of Per2:luc and *Cry1*-luc bioluminescence (mean ± SEM, n = 5–13) from SCN transduced with LV:Syn-hM3DGq-IRESmCherry. Addition of Gq stimulation by CNO significantly increased period (p < 0.01, two-way ANOVA with Bonferroni correction) and decreased the rhythms’ amplitude (two-tailed t test). (C and E) Left: Representative Per2:luc and *Cry1*-luc SCN transduced with LV:Syn-hM3DGq-IRESmCherry and AAV:Syn-GCaMP3. Syn-hM3DGq-IRESmCherry^+^ cells constituted only a relatively small fraction of the neurons within the cultures (Per2:luc: DREADD^+^ 155 ± 21 n = 3; *Cry1*-luc: DREADD^+^ 134 ± 31 n = 3), when compared to GCaMP3^+^, Per2^+^ and *Cry1*^+^. Right: Addition of CNO alters GCaMP3, Per2:luc and *Cry1*-luc oscillations. (D and F) Representative serial images and Poincaré plots depict the progression of the center of luminescence (CoL, white spot) for 3 days before (gray plots, left panels) and after CNO addition (red plots, right panels). Both reporters consistently showed a reduction in the dorsomedial dynamics of the CoL. Plots are representative of at least 3 SCN for each reporter. Scale bars: 50 μm. See also Movies S7 and S8.

DREADD-mediated activation of Gq signaling elicited major changes in the structure of [Ca^2+^]_i_, CRE and *Per1* circadian rhythms, suggesting a global reorganization of the time-encoding properties of the SCN that involved both the intracellular and intercellular specification of time. To determine how far this reorganization extended, we focused on the downstream events of the previously characterized circadian program. SCN expressing Per2:luc or *Cry1*-luc reporters were transduced with LV:Syn-hM3DGq-IRESmCherry (Figure 6). These genetically encoded reporters signal different components of the TTFL: posttranslational and E-box-mediated transcription, respectively, allowing for analysis of the entire SCN circuit. As with *Per1*-luc, Gq activation consistently lengthened period and reduced the amplitude of both Per2:luc protein oscillations and *Cry1*-dependent transcription (Figures 6A and 6B). Moreover, whereas Gq activation increased Per2:luc baseline levels, consistent with activation via CREs, the *Cry1*-luc baseline was reduced, likely reflecting the negative regulatory influence on its E boxes of (unobserved) increased Per protein abundance. Consistent with this, Gq-mediated effects upon *Cry1*-luc were delayed until the second cycle after treatment, as the consequences of Gq stimulation cascaded through the circadian program.

Thus, Gq activation consistently reduced amplitude and lengthened period to ∼25 hr of CRE, *Per1*, Per2, and *Cry1* rhythms, even though the chimeric SCN had a minority of DREADD-expressing neurons. Analysis of [Ca^2+^]_i_ cellular oscillations revealed, however, that the change in the aggregate period could not be ascribed to a coherent shift of all cells toward a 25 hr period, but rather to a pronounced period scatter, trending toward longer periods and likely reflecting weakened internal coupling (Figure 5). The consistency of the ∼25 hr rhythmicity tracked by all reporters and the stability of the oscillations, led us to hypothesize that Gq stimulation triggers a profound, but specific rearrangement of the spatiotemporal dynamics of the circadian oscillations, rather than a general loss of coherence within the circuit. To test this, the spatiotemporal dynamics of Per2:luc and *Cry1*-luc expression were analyzed. To standardize analysis of the spatiotemporal wave of gene expression, a cumulative geometric measure was computed: the center of luminescence (CoL), defined as the center of mass of the distributed bioluminescence signal emanating from the SCN (see Experimental Procedures). The x and y coordinates of the CoL were determined in each frame and their evolution plotted before and during Gq activation. CoL positions were highly conserved and reproducible between and within slices, defining the specific and stereotypical circuit-level organization of the SCN. Upon Gq-pathway stimulation, [Ca^2+^]_i_ circadian rhythms were dysregulated, as noted earlier (Figures 6C and 6E). Moreover, the spatial dynamics of the CoL of both Per2 and *Cry1* were affected in a common way: they were compressed and displaced ventrally to a new steady-state oscillation (Figure 6D and 6F; Movies S7 and S8). This change was not stochastic: rather it was directed and reflected a specific reduction of the TTFL signal within the dorsomedial SCN.

### Reprogramming by Gq Signaling Requires Intrinsic VIPergic Coupling within the SCN Network

**Figure 7:**
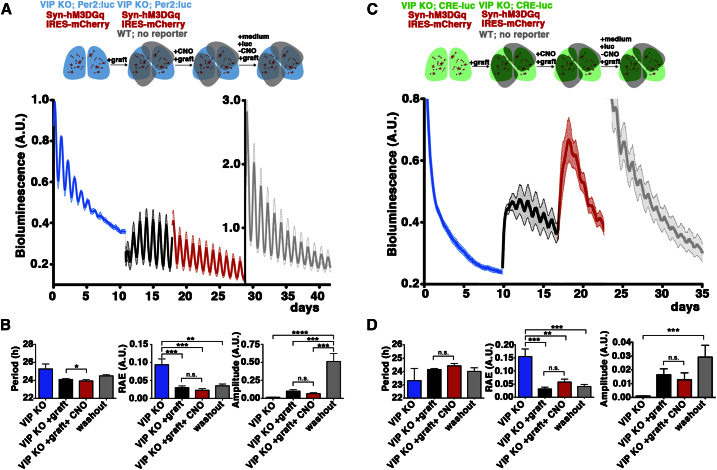
Reprogramming the Pacemaker by Gq Signaling Requires the Intrinsic, VIP-Mediated Coupling of the SCN Network (A) Group plots of bioluminescence from VIP^−/−^, Per2:luc SCN transduced with LV:Syn-hM3DGq-IRESmCherry. Note weak, damping oscillations (blue plots) restored by WT SCN graft (black plots) and no effect exerted by Gq activation (red plot) before and after CNO washout (gray plot). (B) Group data showing that activation of Gq signaling by CNO (100 nM) did not affect period, amplitude or robustness, neither in the presence of CNO (red plots), nor after washout (gray plots). (C and D) As for (A) and (B) but with SCN transduced with LV:CRE-luc. Grafting caused an immediate increase in baseline and rhythm of CRE activity. Activation of DREADD-mediated Gq by CNO (100 nM) (red), acutely induced CRE but no effects on circadian properties were observed. (All data mean + SEM, n = 5; period: two-way ANOVA, amplitude and RAE ratios: two-tailed t test.) See also Figure S3.

The ability of a minority of neurons with activated Gq signaling to re-program the SCN circuit presumably depends upon intercellular coupling involving both [Ca^2+^]_i_ and CRE activation. Having shown the importance of VIP in sustaining the overall levels and network coherence of both CRE activation and intracellular calcium (Figures 1E–1G and 4C–4E), we hypothesized that intrinsic VIPergic signaling may be necessary to transmit the effects of Gq activation across the network. To test this directly we developed a protocol in which SCN from VIP^−/−^ Per2:luc mice were first transduced with the Gq DREADD. As anticipated, PMT recordings revealed a rapid damping of circadian bioluminescence due to VIP insufficiency (Figures 7A and 7B). Reporter- and DREADD-free, WT SCN was then grafted onto the mutant SCN to test its sensitivity to graft-derived VIP (and other) cues (Maywood et al., 2011). The grafts restored robust, high amplitude circadian rhythms in the VIP-deficient host. Thus, although the VIP null network is inherently incapable of generating persistent rhythms, the intracellular clock of the VIP^−/−^ cells is still competent to oscillate when provided with suitable exogenous cues. This made it possible to ask whether activation of Gq signaling in the host SCN would reprogram the host SCN circuit and thereby interfere with the exogenously driven oscillation. When CNO was added, circadian oscillation of Per2:luc reporter in the VIP^−/−^ SCN was not affected: i.e., none of the Gq-mediated effects on period, coherence and amplitude noted above were observed (Figures 7A and 7B). To confirm that DREADD/CNO treatment was active in this configuration, VIP^−/−^ slices expressing the LV:CRE-luc reporter instead of Per2:luc were used (Figures 7C and 7D). Again, their damped rhythm was restored by WT SCN grafts, revealing a direct link between transneuronal cues and circadian control over the activation of CREs. Importantly, upon CNO addition there was a marked activation of CRE, thereby confirming effective DREADD/CNO manipulation in the host SCN. As with the Per2:luc report, however, Gq activation did not affect the properties of the CRE rhythms: the host SCN continued to oscillate with no change in period, amplitude or coherence (Figures 7C and 7D). These data demonstrate that Gq-dependent reprogramming of circadian pacemaking within the SCN circuit is effected via the intrinsic intercellular coupling of the network. When the network lacks VIP-mediated coupling and its rhythms are being driven by exogenous cues, local cellular activation of Gq signaling cannot ramify through the circuit and modify its function (Figure S3).

### Direct Activation of Gq Signaling in VIPergic Neurons Mirrors the Circuit Reprogramming Elicited by Untargeted Gq Activation

**Figure 8:**
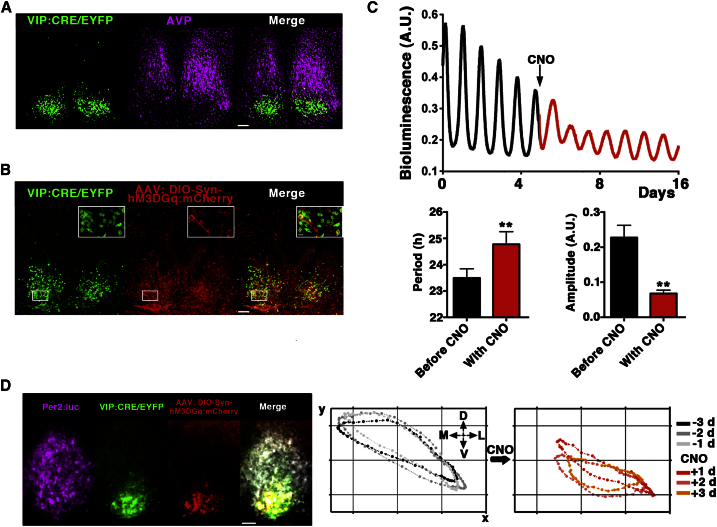
Direct Activation of Gq Signaling in VIPergic Neurons Mirrors the Circuit Reprogramming Elicited by Untargeted Gq Activation (A) Representative microphotographs from *VIP:IRESCre*^*+*^*/EYFP*^*+*^ SCN cultures stained with polyclonal AVP antiserum, delineating a clear core/shell anatomical partitioning. (B) Representative microphotographs from VIP-IRESCre^+^/ EYFP^+^ SCN slices transduced with AAV:DIO-Syn-hM3DGq:mCherry vectors. hM3DGq:mCherry receptor fusions are localized in the plasma membrane (see insets) of VIP:Cre/EYFP^+^ neurons, thus confirming both effective and specific targeting of the VIPergic SCN subpopulation. (C) Representative trace of Per2:luc^+^/VIP:IRESCre^+^ SCN slices transduced with DIO-Syn-hM3DGq:mCherry^+^ and activated by 100 nM CNO. CNO addition increased the baseline, lengthened the period (1.3 ± 0.3 hr) and reduced amplitude of the oscillations (mean ± SEM, n = 7 p < 0.01 paired two-tailed t test). (D) Microphotographs from representative VIP:IRESCre^+^/EYFP^+^ SCN slice transduced with DIO-Syn-hM3DGq:mCherry. Gq signaling was activated by CNO and network effects analyzed by CoL analysis (right panels), showing dorsomedial compression of Per2:luc dynamics. Plots are representative of at least 3 SCN for each reporter. Scale bars: 50 μm. Inset: 10 μm. See also Movie S9.

Activating Gq signaling in a minority of neurons modified the TTFL spatiotemporal dynamic to a new specific state, dependent upon intrinsic VIPergic connectivity. To examine directly the role of VIP neurons in reprogramming we applied an intersectional genetic approach using knockin mice expressing a *VIP:IRESCre* recombinase cassette (Taniguchi et al., 2011) to activate Gq signaling specifically in VIP neurons within the SCN (Figure 8). To delineate their anatomical specificity, *VIP:IRESCre* mice were crossed to *R26-floxed STOP-EYFP*^*+*^ reporter mice and as expected, EYFP expression in the SCN of intercrossed mice (referred to as VIP:Cre/EYFP) was restricted to the core region in the SCN, whereas staining against the Arginine-Vasopressin neuropeptide (AVP) delineated the complementary shell region. This confirmed both effective targeting to the VIP subpopulation and well preserved cytoarchitecture in our SCN slices (Figure 8A). We then transduced these cultures with AAV:DIO-Syn-hM3DGq:mCherry vectors to activate the receptor expression specifically in VIP cells. The hM3DGq:mCherry fusion cassette in DIO (*Double-floxed Inverted Orientation*) vectors is inverted and framed between a double pair of heterotypic antiparallel lox-P sites. Consequently, the cassette is silent, unless expression of Cre recombinase (restricted to VIP neurons in our experiments) flips and activates it (Figure 8B; Movie S9). By transducing Per2:luc^+^/ VIP:Cre/EYFP^+^ SCN cultures with DIO-Syn-hM3DGq:mCherry vectors it was therefore possible to follow the effects on the circadian clock of Gq activation directly targeted to VIP neurons. The effects of this selective activation mirrored those of untargeted Gq stimulation, the Per2:luc oscillations showing increased baseline, prolonged period and reduced amplitude in the presence of CNO (Figure 8C). Moreover, CoL analysis showed that the spatiotemporal dynamics of Per2:luc circadian expression were compressed and displaced ventrolaterally (Figure 8D), a reorganization of the network comparable to that observed following untargeted Gq stimulation. These data demonstrate for the first time that Gq activation in VIP neurons alone is sufficient to reprogram circuit circadian timing, thus revealing the existence of a Gq-Ca^2+^-VIP loop that mediates network control of SCN circadian time.

## Discussion

To interrogate the relationship between G-coupled pathways, cytosolic signaling and the transcriptional feedback loops at the core of the SCN circadian pacemaker, we have developed a combination of viral transduction and multimodal, real-time imaging to map the circadian phase landscape of the SCN. This revealed the orderly circadian progression of cytosolic signaling events ([Ca^2+^]_i_ and CRE activation) in relation to the TTFL components (*Per1*, Per2, and *Cry1*) that together define circadian time. We then tested the role of individual G-coupled signaling pathways, upstream of [Ca^2+^]_i_ and CRE activation, in specifying this circadian program by using DREADD pharmacogenetic receptors to manipulate individual G-coupled cascades. This revealed selective and particular roles for Gs, Gi and Gq in circadian programming. Although Gs and Gi were both able to temporarily modify CRE dynamics, their effects on circadian pacemaking were reversible. In contrast, Gq activation in a minority of cells permanently reprogrammed the spatiotemporal dynamic of intracellular calcium and TTFL components across the SCN. Our data thus not only reveal the complexity of the intrinsic temporal structure of the SCN pacemaker, but also highlight a fundamental role exerted by pathways regulating intracellular calcium in determining the intrinsic properties of the SCN circuit. In particular, the Gq-[Ca^2+^]_i_ axis assumes a prominent role in defining the circadian program, as shown by the major reprogramming of the network achieved by Gq stimulation in a minority of neurons. Importantly, this reorganization requires the intrinsic VIPergic interneuronal coupling in order for it to be broadcast to the rest of the network, and its activation in VIP neurons alone is sufficient to reprogram the circuit, thus revealing a Gq-[Ca^2+^]_i_-VIP *leitmotif* that determines the intrinsic network coherence of the SCN pacemaker.

Dependence of circadian pacemaking on intracellular calcium has been indicated in both mammals (Lundkvist et al., 2005) and *Drosophila* (Harrisingh et al., 2007), although the mechanisms and patterns of [Ca^2+^]_I_ are poorly understood. Key to our approach, therefore, was reliable long-term real-time monitoring of [Ca^2+^]_i_ and CRE activation. The AAV:Syn-GCaMP3 reporter (Tian et al., 2009, 2012) offered us unprecedented sensitivity, utility and stability of [Ca^2+^]_i_ recording when compared to FRET-based reporters (Enoki et al., 2012; Hong et al., 2012; Ikeda et al., 2003). Equally the LV:CRE-luc reporter provided circadian recordings of comparable quality, well beyond those previously achieved (O’Neill et al., 2008; Obrietan et al., 1999). Interestingly, the cellular and aggregate circadian profiles of both the cytosolic reporters were highly asymmetrical and contrasted markedly with the sinusoidal pattern of TTFL transcriptional reporters. These profiles are consistent with a model in which, rather than a progressive waxing and waning, SCN neurons experience an acute burst of [Ca^2+^]_i_ and CRE activation once every cycle. Such bursts may represent a digital trigger to more precisely time the high-amplitude “analog” rhythm of their target genes in the TTFL. By combination with Per2:luc bioluminescence, we mapped the peak of [Ca^2+^]_i_ to CT07, which is coincident with elevated cAMP levels (Doi et al., 2011; O’Neill et al., 2008) and electrical firing rates (Atkinson et al., 2011). Activation of CREs was maximal soon after these peaks, and was highly dependent on electrical activity within the circuit, damping rapidly in the presence of TTX (Figure 1D). Our phase-map therefore reveals a sequence in which increased electrical firing sustains increased [Ca^2+^]_i_, which in turn drives a circadian surge in CRE activation (likely facilitated cooperatively by raised cAMP levels, a canonical property of CRE-mediated gene expression (Shaywitz and Greenberg, 1999)). A comparable relationship between enhanced electrical firing and elevated CRE-dependent gene expression was reported in *Drosophila* “clock” neurons (Mizrak et al., 2012), suggesting that electrical-genetic coupling is a conserved property of neuronal pacemakers that underlies their greater precision in comparison to peripheral oscillators. Thus, upstream cytosolic signaling events mediated by cAMP and [Ca^2+^]_i_ can gain access to transcriptional components of the core pacemaker through the acute circadian surge in CRE activation. In our phase-map *Per1*, which carries numerous CREs, was expressed with an ∼1 hr delay relative to CREs. In contrast, circadian expression of *Cry1*-luc, which lacks CREs and relies on E boxes for circadian expression, was only initiated 3 hr after *Per1*. This raises the possibility that cytosolic signals acting via CRE not only sustain the TTFL but also confer specific phases to its component parts, thereby determining its internal structure.

G-coupled receptor signaling is necessary to sustain both firing rates (Aton et al., 2005; Cutler et al., 2003) and cytosolic [Ca^2+^]_i_ and CRE oscillations (Figures 1E–1G and 4C–4E) in the SCN, making it an ideal hub to integrate network events to the TTFL intracellular clockwork, via cytosolic rhythms. To determine the specific contributions of different G signaling pathways in defining SCN circuit properties, we manipulated Gs, Gq and Gi individually, creating a functional discontinuity within the network by using LV delivery of DREADD receptors to a minority of SCN neurons (≪ 40%) (Armbruster et al., 2007; Rogan and Roth, 2011). Importantly, this occurred in otherwise genetically intact SCN circuits, not subject to the developmental confounds associated with germ-line mutations or embryonic chimerism (Ko et al., 2010; Low-Zeddies and Takahashi, 2001). The acute effects of Gs, Gi, and Gq on CRE activation confirmed their potential to affect the circadian program, but Gs- and Gi-elicited effects were reversible. The longer-term and irreversible effects of Gq activation, however, were striking: lengthening the period of circadian CRE activation and suppressing rhythm amplitude. Moreover, single-cell analysis showed that Gq-mediated activation of CREs occurred sequentially in transduced and nontransduced cells, thereby reprogramming cellular behavior across the network. This reprogramming markedly affected the *Per1*, Per2 and *Cry1* rhythms: in particular Gq activation dysregulated the spatiotemporal wave of gene activation in Per2:luc and *Cry1*-luc SCN. The phase-leading activation of TTFL elements in the dorsomedial SCN was reduced and the wave contracted to a tighter, more ventrally located trajectory. Thus, pharmacogenetic activation of Gq signaling in a minority of SCN neurons reverberated across the SCN circuit to establish a new steady-state of ensemble activity, defined by a 25h period, reduced amplitude and spatiotemporal reorganization. The origin of this reprogramming likely resides in altered [Ca^2+^]_i_ dynamics: whereas Gs- or Gi-dependent signaling had no lasting effect on [Ca^2+^]_i_ rhythms, activation of Gq reprogrammed [Ca^2+^]_i_ rhythms, dispersing cell phases and suppressing rhythm amplitude across the SCN.

We hypothesized that the intrinsic VIP signaling (An et al., 2011; Dickson and Finlayson, 2009) may account for the Gq-[Ca^2+^]_i_ mediated reprogramming of the SCN network. Our SCN grafting paradigm (Maywood et al., 2011) showed that activation of Gq signaling in the VIP null host SCN could acutely induce CRE-luc, but was unable to modify on-going circadian rhythms of Per2:Luc expression. Thus, reprogramming by Gq requires competent intrinsic VIPergic signaling, suggesting that pathways addressed via Gq DREADD are responsible for maintaining the stereotypical spatiotemporal program of the wild-type SCN. Having demonstrated that intrinsic VIPergic signaling is *necessary* to mediate the effects of Gq on the SCN circuit, we then tested if stimulation of Gq in VIP neurons was *sufficient* to reproduce the same effects. By exploiting an intersectional genetics approach, we found that activation of Gq in VIP cells alone was indeed sufficient for reprogramming. These results therefore frame the observed phenotypes in a more physiological context. The VIPergic subpopulation is a well defined retinorecipient subpopulation of the SCN, and so it is tempting to speculate that the Gq-VIP axis may mediate the fundamental effects of the light-dark cycle on entrainment and photoperiodic adaptation of the SCN network (Inagaki et al., 2007).

In conclusion, previous studies have emphasized the intrinsic resistance of the SCN tissue to genetic manipulations of TTFL components (Butler and Silver, 2009; Liu et al., 2007). In contrast, our study has unveiled an unforeseen plasticity of the SCN as a circuit, able quickly to modify its time-encoding properties after intracellular manipulations of only a few cells. Importantly, whether the viral transduction was not knowingly targeted to particular neuronal types or specifically directed at VIP neurons, it consistently reprogrammed the spatiotemporal dynamics of the circuit to a specific state, and this effect was consistent across various cytosolic and transcriptional reporters. Thus, activation of Gq signaling within a minority of SCN neurons was sufficient to reprogram the ensemble function, but only in the presence of intrinsic VIPergic transmission. In contrast to the assumed genetic robustness and resilience, therefore, the SCN network displays sensitive and adaptable circuitry, reorganizing in the light of changes in individual cellular behavior. Our data are therefore consistent with recent theoretical considerations of the SCN circuit suggesting that a source of as few as 25 cells can be sufficient to determine the temporal properties of the entire network (Kori et al., 2012). The current findings highlight the role of VIP neurons in defining canonical circadian properties, such as period and amplitude. Our intersectional and imaging approach has thus started to address unresolved issues surrounding the relative contributions of individual neurons, neuronal subpopulations and network-encoded properties to the specification of circuit-level circadian behavior (Mohawk and Takahashi, 2011).

## Experimental Procedures

### Animals

All procedures were conducted under the UK Animals (Scientific Procedures) Act, 1986, approved by Home Office licenses and local ethical review. Per2:luc, Vip^−/−^ and Fbxl3^Afh/Afh^ mice were provided, respectively, Dr. J. Takahashi (UTSW Medical Center, Dallas), Dr. C. Colwell (UCLA), and Dr. P. Nolan (MRC, Harwell, UK). *Cry1*-luc mice were generated in-house using a previously validated *Cry1*-luc plasmid (Fustin et al., 2009). VIP-IRESCre (Viptm1(cre)Zjh/J) and R26 floxed STOP EYFP (B6.129X1-Gt(ROSA)26Sortm1(EYFP)Cos/J) mice were obtained from the Jackson Laboratory (Bar Harbor, Maine, USA).

### Viral Transduction of SCN Slice Cultures

LVs produced as previously described (Brancaccio et al., 2010). AAV2/1:Syn-GCaMP3-WPRE from Penn Vector Core (Loren L. Looger, Ph.D., and Janelia Farm Research Campus, HHMI). AAV2:DIO-Syn-hM3DGq:mCherry from UNC Vector Core. Organotypic SCN cultures from p4–p6 WT or mutant mice prepared as described (Maywood et al., 2006). Two days after the dissection concentrated LV particles were dropped directly onto the slice. For GCaMP3 experiments SCN slices, previously LV-transduced were super-transduced by dropping the AAVs onto the slice.

### Pharmacogenetic Manipulations of WT and VIP^−/−^ Grafted SCN Slices

SCN slices transduced by LV encoded DREADDs presented with 100nM Clozapine-N-Oxide (CNO) (in ddH_2_O) (Enzo Life Sciences). Vehicle treated SCN received ddH_2_O as vehicle treatment. For washing out, SCN cultures washed four times in HEPES buffered medium and transferred to fresh medium containing 100 nM luciferin (Promega) and the recording restarted immediately afterward. Grafting experiments as elsewhere described (Maywood et al., 2011).

### Data Analysis and Statistical Tests

PMT data analyzed in BioDare (A. Millar, T. Zielinski, University of Edinburgh) by FFT-NLLS. Period, amplitude and relative amplitude error (RAE) calculated on time series lasting ≥5 days. Periods between vehicle and CNO treated SCN slices before/with drug were assessed by repeated-measures two-way ANOVA, with Bonferroni correction. Amplitude ratios (with/before drug treatment) calculated for each sample and statistical significance evaluated by two-tailed t test. Different phases of the grafting experiments were compared by repeated-measures ANOVA with Bonferroni. For single-cell analyses, period, amplitude and RAE values for each oscillator analyzed in BioDare and the Igor pro (WaveMetrics) routine SARFIA (Dorostkar et al., 2010). Statistical significance evaluated by Kolmogorov-Smirnov test. Median and aadm values determined as measurements of average and variance, respectively. For synchrony analysis, data analyzed in Oriana 4 (KCS, UK). For center of luminescence (CoL) analysis bioluminescence levels from a single SCN were normalized and thresholded. Time series were then analyzed in Igor Pro by an in-house designed plug-in to determine the center of mass X and Y coordinates within each frame (time resolution, 30 min) and represented in Poincarè plots 3 days before CNO addition and in the 3 days with CNO. Data analyses and statistical tests performed in Excel:mac 2011 (Microsoft) and Prism 5 (GraphPad). Graphs prepared in Prism.

See Supplemental Experimental Procedures for more details.

## Supplemental Information

Document S1. Figures S1–S3, Table S1, and Supplemental Experimental Procedures

Movie S1. Representative Time-lapse Video Recording of a Wild-Type SCN Organotypic Slice Transduced by the LV:CRE-luc LUT code: magenta hot. See also Figure 1.

Movie S2. Representative Time-Lapse Video Recording of a Wild-Type SCN Organotypic Slice Transduced by the AAV:Syn-GCaMP3 LUT code: thallium. See also Figure 4.

Movie S3. Representative Time-Lapse Video Recording of Per2:luc SCN Slice Transduced by the AAV:Syn-GCaMP3 LUT code: Syn-GCaMP3 = green; Per2:luc = magenta. See also Figure 4.

Movie S4. Representative Time-Lapse Video Recording of a Pharmacogenetic Gq Activation Experiment Wild-type SCN slice transduced by AAV:Syn-GCaMP3 and LV:hM3DGq-IRESmCherry was recorded before CNO addition, in the presence of CNO and after washing out. CNO addition is marked on the movie. LUT code: Syn-GCaMP3 = green; LV:hM3DGq-IRESmCherry = red. See also Figure 5.

Movie S5. Representative Video Time-Lapse Recording of a Pharmacogenetic Gs Activation Experiment Wild-type SCN slice transduced by AAV:Syn-GCaMP3 and LV:Syn-rM3/β1Gs-IRESmCherry was recorded before CNO addition, in the presence of CNO and after washing out. CNO addition is marked on the movie. LUT code: Syn-GCaMP3 = green; LV:Syn-rM3/β1Gs-IRESmCherry = red. See also Figure 5.

Movie S6. Representative Time-Lapse Video Recording of a Pharmacogenetic Gi Activation Experiment AAV:Syn-GCaMP3 and LV:hM4DGi-IRESmCherry was recorded before CNO addition, in the presence of CNO and after washing out. CNO presence is marked on the movie. LUT code: Syn-GCaMP3 = green; LV:hM4DGi-IRESmCherry = red. See also Figure 5.

Movie S7. Representative Time-Lapse Video Recording of the Effects of Pharmacogenetic Gq Activation on the Per2:luc Center of Luminescence (CoL) Per2:luc SCN slice transduced by the LV:hM3DGq-IRESmCherry was recorded before CNO addition and in the presence of CNO. CNO presence is marked on the movie. Per2:luc = magenta; CoL = white. See also Figure 6.

Movie S8. Representative Time-Lapse Video Recording of the Effects of Pharmacogenetic Gq Activation on the *Cry1*-luc Center of Luminescence (CoL) *Cry1*-luc SCN slice transduced by the LV:Syn-hM3DGq-IRESmCherry was recorded before CNO addition and in the presence of CNO. CNO presence is marked on the movie. *Cry1*-luc = magenta; CoL = white. See also Figure 6.

Movie S9. Representative Stack from SCN Slices from VIP:Cre/EYFP^+^ Mice, Transduced with AAV:DIO-Syn-hM3DGq:mCherry Note the high rate of viral transduction, as well as the specific expression of the receptor fusion in the plasma membrane of VIP positive neurons. VIPCre/EYFP^+^ = green; hM3DGq:mCherry: red. Related to Figure 8.
