# Supplementary material for: A Gq-Ca2+ Axis Controls Circuit-Level Encoding of Circadian Time in the Suprachiasmatic Nucleus
Source: Neuron. 2013 May 22;78(4):714–28. doi: 10.1016/j.neuron.2013.03.011 (PMC3666084; doi:10.1016/j.neuron.2013.03.011)
Supplement: Document S1. Figures S1–S3, Table S1, and Supplemental Experimental Procedures [file mmc1.pdf]

Neuron, Volume *78*

Supplemental Information

A Gq-Ca^{2+} Axis Controls Circuit-Level Encoding

of Circadian Time in the Suprachiasmatic Nucleus

Marco Brancaccio, Elizabeth S. Maywood, Johanna E. Chesham, Andrew S.I. Loudon, and Michael H. Hastings

Inventory of Supplemental Information

1. **Figure S1.** Gq effects on circadian properties of CRE activation in SCN cultures are dose- and time-dependent, and persist after drug removal. Related to Figure 2.
2. **Figure S2.** Forskolin/IBMX treatment dramatically activates CREs without altering the circadian properties of the SCN**.** Related to Figure 5.
3. **Figure S3.** A model for Gq-mediated network control of the time-encoding properties of the SCN**.** Related to Figure 7.
4. **Table S1.** Summary of the circadian properties in GCaMP3 oscillators for representative Syn-hM3DGq-mCherry, Syn-rM3/β1Gs-mCherry, Syn-hM4DGi-mCherry treated SCN cultures. Related to Figure 5.
5. **Movie S1:** Representative time-lapse video recording of a wild type SCN organotypic slice transduced by the LV:CRE-luc. Related to Figure 1.
6. **Movie S2:** Representative time-lapse video recording of a wild type SCN organotypic slice transduced by the AAV:Syn-GCaMP3. Related to Figure 4.
7. **Movie S3:** Representative time-lapse video recording of Per2:luc SCN slice transduced by the AAV:Syn-GCaMP3. Related to Figure 4.
8. **Movie S4:** Representative time-lapse video recording of a pharmacogenetic Gq activation experiment. Related to Figure 5.
9. **Movie S5:** Representative video-time lapse recording of a pharmacogenetic Gs activation experiment. Related to Figure 5.
10. **Movie S6:** Representative time-lapse video recording of a pharmacogenetic Gi activation experiment. Related to Figure 5.
11. **Movie S7:** Representative time-lapse video recording of the effects of pharmacogenetic Gq activation on the Per2:luc Center of Luminescence. Related to Figure 6
12. **Movie S8:** Representative time-lapse video recording of the effects of pharmacogenetic Gq activation on the *Cry1*-luc Center of Luminescence (CoL). Related to Figure 6
13. **Movie S9:** Representative stack from SCN slices from VIP:Cre/$EYFP^{+}$ mice, transduced with AAV:DIO-Syn-hM3DGq:mCherry. Related to Figure 8
14. **Supplemental Experimental Procedures**
15. **Supplemental References**

Supplemental Information

Supplemental Figures

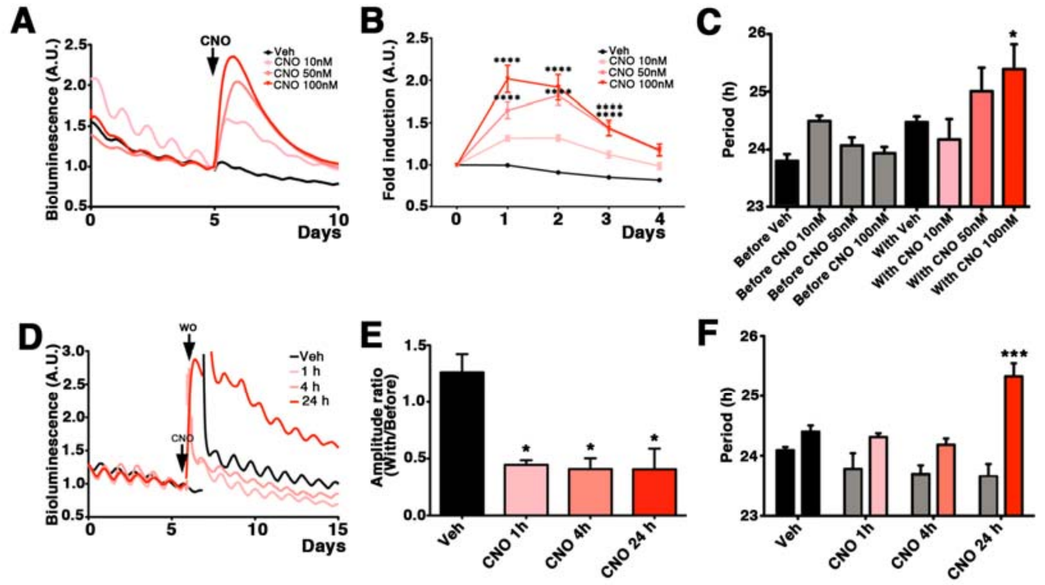

Figure S1. Gq effects on circadian properties of CRE activation in SCN cultures are dose- and time-dependent, and persist after drug removal. Related to Figure 2.

(A) Mean traces of bioluminescence recording from SCN cultures transduced with LV:Syn-hM3DGq-IRESmCherry and LV:CRE-luc reporters and treated with CNO at 10nM, 50nM or 100nM. (B) Baseline activity of CRE changed dose-dependently and reached its maximum after 1 day of treatment at the highest concentration tested (100nM). (C) Period of CRE rhythms in LV:Syn-hM3DGq-IRESmCherry/LV:CRE-luc treated slices was lengthened by increasing CNO concentrations, reaching statistical significance at 100nM CNO ($p<0.05$). (D). Representative traces of PMT recordings from SCN transduced with LV:Syn-hM3DGq-IRESmCherry and LV:CRE-luc vectors and treated with CNO (100nM) for various time intervals (1h, 4h, 24h) before drug washout. Although washing out of CNO effectively interrupted the CRE surge, the circadian effects exerted by Gq-pathway activation persisted after drug removal. In particular, 1 h of treatment was sufficient to decrease significantly

the amplitude of the oscillation (E), whereas 1 day of treatment was necessary to increase period length (F) ($p<0.001$). All data Mean+SEM $n \geq 3$. Period stats: 2-ways ANOVA with Bonferroni correction, amplitude and RAE ratios: ANOVA with Bonferroni correction).

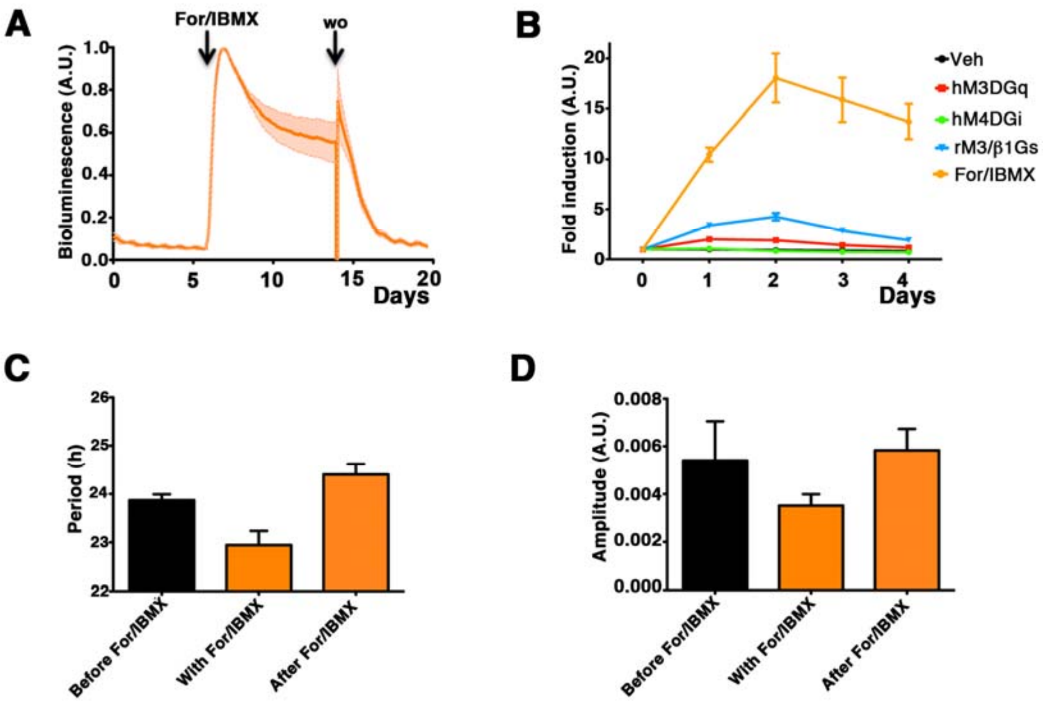

Figure S2. Forskolin/IBMX treatment dramatically activates CREs without altering the circadian properties of the SCN. Related to Figure 5.

(A) Mean bioluminescence plot from SCN transduced with the LV:CRE-luc reporter and treated with forskolin/IBMX (20μM/ 500μM) to increase intracellular cAMP concentrations. Note immediate burst of CRE activation, sustained until the drugs were washed out after 8 days. After for/IBMX removal the CRE levels returned back to initial values. (B) Comparison between baseline CRE activation induced by forskolin/IBMX treatment and CNO treatment with the three DREADD configurations. Although CRE levels were induced to a much higher level (~17x) and for a longer time (8 days) by forskolin/IBMX treatment, when compared to hM3DGq or rM3/β1Gs CNO treated cultures, no significant period or amplitude change was observed during or after the drug treatment. (C, D) Group data for circadian period and amplitude of SCN treated with forskolin/IBMX. Note that sustained high levels of CRE activation were not detrimental for circadian oscillations in SCN and were not, *per se*, predictive of irreversible changes in circadian properties**.** All data Mean+SEM; period and amplitude: ANOVA with Bonferroni correction).

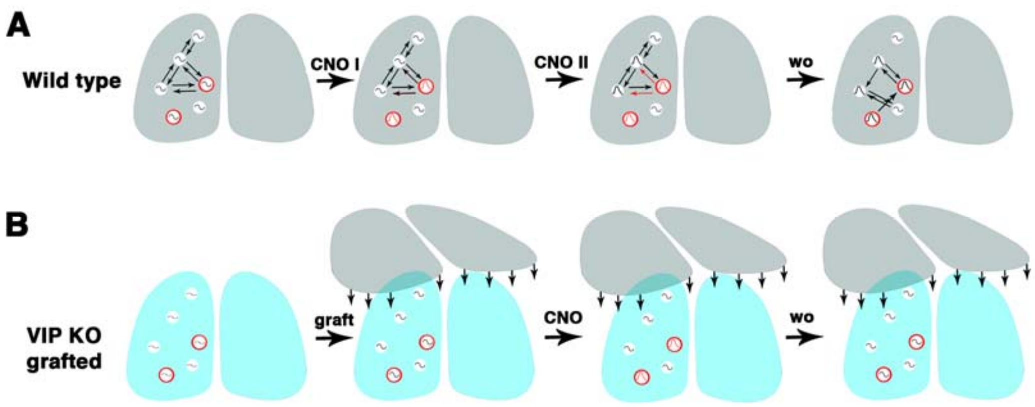

Figure S3. A model for Gq-mediated network control of the time-encoding properties of the SCN. Related to Figure 7.

(A) In wild type SCN, neurons expressing the Syn-hM3DGq-mCherry (red circles) show normal circadian oscillations. CNO addition stimulates Gq signalling in neurons expressing the DREADD receptors (CNO I step), as revealed by CRE activation in those cells. After a delay, the DREADD-activated neurons trigger a response in the surrounding neurons (white circles) that relies on the intercellular connectivity of the network (red arrows) (CNO II step). Spreading of the signal across the network changes the circadian properties of the single oscillators and specifically lengthens the period encoded by the network as a whole, presumably through an irreversible reorganization of its intrinsic coupling properties (wo step). (B) In VIP-null SCN the coupling of the network is deficient and the absence of intercellular signals feeding into CRE weakens the strength of the intracellular TTFL loop, leading to a population of poorly rhythmic and poorly synchronized oscillators. Grafting VIP-null SCN with WT SCN restores the rhythmicity because of the circadian secretion of VIP (and other peptides), which provides exogenous stimulation of CRE and thereby reactivates the intracellular TTFL machinery, (graft step). Addition of CNO to these co-cultures is still able to trigger the CRE response in neurons transduced by hM3DGq (CNO step). However, in these conditions the circadian rhythm of the oscillators is not dependent on the intrinsic coupling of the network, and in the absence of that

coupling stimulation of Gq signalling cannot re-program the time-encoding properties of the network.

Supplemental Tables

Table S1. Summary of the circadian properties in GCaMP3 oscillators for representative Syn-hM3DGq-mCherry, Syn-rM3/β1Gs-mCherry, Syn-hM4DGi-mCherry treated SCN cultures. Related to Figure 5.

Syn-hM3DGq-mCherry*				
GCaMP3				
	Before CNO	With CNO	After CNO	KS-test
Period (hs)	23.77+0.17	23.67+0.90	24.46+1.19	p<0.0001** , p<0.0001***
Amplitude (A.U.)	10.24+5.01	7.33+4.27	4.48+2.55	p<0.0001, p<0.0001
RAE (A.U.)	0.06+0.03	0.11+0.05	0.15+0.08	p<0.0001, p<0.0001
r ****	0.88	0.21	0.023	-
n	181	181	181	

Syn-rM3/β1Gs-mCherry				
GCaMP3				
	Before CNO	With CNO	After CNO	KS-test
Period (hs)	23.38+0.11	24.02+0.28	24.03+0.31	p<0.0001, p<0.0001
Amplitude (A.U.)	123+49.3	121.1+57.1	160.7+67.9	ns,p<0.0001
RAE (A.U.)	0.07+0.03	0.07+0.06	0.07+0.05	p<0.0001, p<0.05
r ****	0.965	0.783	0.573	-
n	174	174	174	

Syn-hM4DGi-mCherry				
GCaMP3				
	Before CNO	With CNO	After CNO	KS-test
Period (hs)	24.53+0.47	23.57+0.80	23.48+0.15	p<0.0001, p<0.0001
Amplitude (A.U.)	497+288	401+232	1141+476	p<0.01,p<0.0001
RAE (A.U.)	0.11+0.04	0.14+0.66	0.06+0.02	p<0.0001, p<0.0001
r ****	0.852	0.192	0.762	-
n	259	259	259	

* All values are median + average absolute distance from the median (aadm)
** Kolgomorov-Smirnov test p (before vs with CNO)
*** Kolgomorov-Smirnov test p(before vs after CNO)
**** r= Length of the mean vector in the Rayleigh plot

SUPPLEMENTAL MOVIES LEGENDS

Movie S1. Representative time-lapse video recording of a wild type SCN organotypic slice transduced by the LV:CRE-luc. LUT code: magenta hot. Related to Figure 1.

Movie S2: Representative time-lapse video recording of a wild type SCN organotypic slice transduced by the AAV:Syn-GCaMP3. LUT code: thallium. Related to Figure 4.

Movie S3: Representative time-lapse video recording of Per2:luc SCN slice transduced by the AAV:Syn-GCaMP3. LUT code: Syn-GCaMP3=green; Per2:luc=magenta. Related to Figure 4.

Movie S4: Representative time-lapse video recording of a pharmacogenetic Gq activation experiment. Wild type SCN slice transduced by AAV:Syn-GCaMP3 and LV:hM3DGq-IRESmCherry was recorded before CNO addition, in the presence of CNO and after washing out. CNO addition is marked on the movie. LUT code: Syn-GCaMP3=green; LV:hM3DGq-IRESmCherry= red. Related to Figure 5.

Movie S5: Representative video-time lapse recording of a pharmacogenetic Gs activation experiment. Wild type SCN slice transduced by AAV:Syn-GCaMP3 and LV:Syn-rM3/β1Gs-IRESmCherry was recorded before CNO addition, in the presence of CNO and after washing out. CNO addition is marked on the movie. LUT code: Syn-GCaMP3=green; LV:Syn-rM3/β1Gs-IRESmCherry = red. Related to Figure 5.

Movie S6: Representative time-lapse video recording of a pharmacogenetic Gi activation experiment. AAV:Syn-GCaMP3 and LV:hM4DGi-IRESmCherry was recorded before CNO addition, in the presence of CNO and after washing

out. CNO presence is marked on the movie. LUT code: Syn-GCaMP3= green; LV:hM4DGi-IRESmCherry = red. Related to Figure 5.

Movie S7: Representative time-lapse video recording of the effects of pharmacogenetic Gq activation on the Per2:luc Center of Luminescence (CoL). Per2:luc SCN slice transduced by the LV:hM3DGq-IRESmCherry was recorded before CNO addition and in the presence of CNO. CNO presence is marked on the movie. Per2:luc=magenta; CoL=white. Related to Figure 6

Movie S8: Representative time-lapse video recording of the effects of pharmacogenetic Gq activation on the *Cry1*-luc Center of Luminescence (CoL). *Cry1*-luc SCN slice transduced by the LV:Syn-hM3DGq-IRESmCherry was recorded before CNO addition and in the presence of CNO. CNO presence is marked on the movie. *Cry1*-luc=magenta; CoL=white. Related to Figure 6

Movie S9: Representative stack from SCN slices from VIP:Cre/EYFP^{+} mice, transduced with AAV:DIO-Syn-hM3DGq:mCherry. Note the high rate of viral transduction, as well as the specific expression of the receptor fusion in the plasma membrane of VIP positive neurons. VIPCre/EYFP^{+}=green; hM3DGq:mCherry: red. Related to Figure 8

SUPPLEMENTAL EXPERIMENTAL PROCEDURES

Molecular biology for DREADD lentiviral transfer vectors

Transfer vector plasmid for LV:CRE-luc was produced by inserting the luciferase reporter from the PGL3-basic (Promega) downstream to the CRE promoter present in the pCRE-DD-ZsGreen1 plasmid (Clontech) and transferring the resulting CRE-luc cassette into a suitable third generation self-inactivating (SIN) transfer vector. Transfer vector plasmids for the pharmacogenetic receptors LV:Syn-hM3DGq-IRESmCherry-WPRE and LV:Syn-hM4DGi-IRESmCherry-WPRE were generated by cloning the hM3DGq-IRESmCherry and hM4DGi-IRESmCherry cassettes (gift from Brian Roth, University of North Carolina, Chapel Hill) into a previously generated Syn-EGFP-WPRE transfer plasmid, downstream of the human synapsin promoter. To produce LV:Syn-rM3/ β1Gs-IRESmCherry-WPRE, first the IRES-mCherry cassette was inserted downstream to the human synapsin promoter in the hSyn-EGFP-WPRE plasmid and then rM3/β1Gs cassette from the pcDNA3.1 rM3/β1Gs-IRESmCitrine (gift from Brian Roth) inserted downstream to the Syn promoter of the newly generated Syn-IRESmCherry plasmid.

Viral transduction of SCN slice cultures extended procedures

Organotypic SCN cultures from p4-p6 WT or mutant mouse pups were prepared as described (Hastings et al., 2005). Two days after the dissection up to 5µl of concentrated LV particles (single or mix preparations, 1-3*10^6 TU/µl) were dropped directly onto the slice. After 2 days, LV conditioned medium was replaced by fresh medium. SCN slices were then transferred into sealed Petri dishes for long-term recording by PMT or imaging by CCD camera (Maywood et al. 2006). For GCaMP3 experiments SCN slices, previously transduced with LV particles (not less than 7 days before) were super-transduced by dropping the AAV particles onto the slice (0.5 µl, 5*10^9

TU/μl). GCaMP3 expression could typically be seen after ~7 days of transduction, with fluorescent levels typically rising during the first weeks. $[Ca^{2+}]_i$ at the nadir and at the zenith were estimated as previously described (Maravall et al., 2000; Tsien, 1989) using the formula $[Ca^{2+}]_i= Kd(f/f_{max}-1/R_f)/(1-f_{max})$, where f is the measured absolute fluorescence, f_{max} is the maximal fluorescence detected by the reporter and R_f is its range (f_{max}/f_{min}). GCaMP3 Kd and R_f are 660nM and 12, respectively (Tian et al., 2012). To determine f_{max}, Syn-AAV-GCaMP3 transduced slices were recorded on CCD for at least two days; afterwards, ionomycin (30μM, Invitrogen) was added to the culture medium and the $[Ca^{2+}]_i$ at the zenith and nadir estimated. For intersectional genetic experiments AAV particles (2.5 μl, $6*10^8$ TU/μl) were directly dropped onto the slice.

Immunofluorescence on SCN cultures

Membrane attached SCN cultures were fixed 1h in PFA 4%, washed 4 times in PBS for 10 minutes and incubated in blocking buffer (FBS 10%, BSA 1%, 0.3% Triton X-100 in PBS) for 3.5 hours. Primary AVP antibody (rabbit polyclonal, Bachem, 1:500) added in blocking mix and incubated O/N at 4 °C and washed out with PBS (4X10’). Slices were then incubated with secondary antibody (anti-rabbit Alexa Fluor 647, Invitrogen, 1:800), washed with PBS (4X10’) and mounted in Vectashield (Vector Labs) plus DAPI for confocal imaging. Native fluorescence was used for detection of Cre driven R26 floxed STOP-EYFP and DIO-hM3DGq:mCherry fusion. Low power stacks were acquired using a Zeiss LSM 710 equipped with a 10X objective. For high-resolution images stacks taken with a 40X oil immersion objective.

Multichannel fluorescence/ bioluminescence time-lapse video microscopy

SCN slices from WT or reporter mouse lines, transduced with LV and/or AAV particles were transferred into sealed glass-bottomed Petri dishes in HEPES buffered medium and imaged by heated stage LV200 Luminoview systems (Olympus), with mounted filter sets for bright field, red and green fluorescence. Bioluminescent and fluorescent signals were collected by Hamamatsu C9100-13 EM-CCD camera. Photomicrographs for each fluorescent and bioluminescent channel were taken every 30 minutes. Time-series were downloaded as grey-scale image stacks and pre-processed in ImageJ (NIH). A false colour LUT was imposed to each channel (LUT code: Bioluminescence reporters= Magenta, GCaMP3= Green, DREADD receptor= Red) and the different channels assembled as composite multi-channel stacks, de-speckled and ROI normalized to subtract background. LUT in Figure 1 and Movies S1 and S2: GCaMP3= thallium; CRE-luc= Magenta hot, for presentation purposes). To compare the phases of different reporters within a single slice the inter-peak distance between the GCaMP3 reporter and the selected bioluminescent reporter was determined by visual inspection. The experiment was repeated ≥3 times for each GCaMP3/bioluminescent reporter pair. For pharmacogenetic experiments, sub-stacks in pre-treatment and after-treatment conditions were manually aligned to allow recognition of single oscillators throughout the entire recording. ImageJ pre-analysed stacks were transferred to Igor Pro (WaveMetrics) and analysed by Semi-Automated Routines for Functional Image Analysis (SARFIA) plugin (Dorostkar et al., 2010). Threshold and pixel limits were set to optimize cell recognition and automated un-biased analysis was performed. Data collected were then processed for further analysis. For co-localisation studies cells expressing CRE:luc reporters were visually identified on the first frame of the recording (conditions blind to red fluorescence signal). Afterwards, red fluorescence (derived from LV:Syn-hM3DGq-IRESmCherry) was assessed and circadian analysis performed on both the CRE-luc^{+}/mCherry^{+} and CRE-luc^{+}/mCherry^{-} sub-populations. For kinetic analysis on CRE induction in CRE-luc^{+}/mCherry^{+} and CRE-luc^{+}/mCherry^{-} subpopulations within the slice, data for each sample were scaled to the minimum (first frame after CNO addition) and plotted.

Mean±SEM were plotted and linear regression performed and deviation from linearity tested (not significant).

Data analysis and statistical tests: extended procedures

PMT data (time resolution: 6 minutes) were normalized before analysis of circadian rhythmicity. CRE-luciferase traces in pharmacogenetic experiments were de-trended with a 24-hour moving average to remove the rising and declining baseline trends elicited by CNO addition. GCaMP3 data were linearly de-trended, to remove the rising baseline trend elicited by sustained activation by the Syn promoter in AAV transduced slices. Data were then analysed in BioDare (A. Millar, T. Zielinski, University of Edinburgh) by FFT-NLLS algorithms. Period, amplitude and relative amplitude error (RAE) were calculated on time series lasting ≥ 5 days. Assessment of statistical significance for period comparison between vehicle and CNO treated SCN slices before/with drug were performed by 2-way ANOVA for repeated measures, with a Bonferroni correction. Amplitude ratios (with/before drug treatment) were calculated for each sample and statistical significance evaluated by 2-tailed t-test. For grafting experiments ANOVA for repeated measures with Bonferroni post-hoc test was performed to compare different phases of the experiment.

For single-cell analyses, data collected by video time-lapse microscopy (time resolution: 30 minutes) of a single SCN area were pre-analysed in ImageJ and SARFIA. ROI manifesting circadian oscillations throughout the time series were analysed in BioDare. Period, amplitude and RAE values for each oscillator were then represented as cumulative frequency distributions and plotted before CNO treatment, in the presence of the drug and after its removal. Statistical significance evaluated by Kolmogorov-Smirnov test (http://www.physics.csbsju.edu/stats/KS-test.n.plot_form.html.). Median and aadm values were determined as measurement of average and variance, respectively.

SUPPLEMENTAL REFERENCES

Hastings, M.H., Reddy, A.B., McMahon, D.G., and Maywood, E.S. (2005). Analysis of circadian mechanisms in the suprachiasmatic nucleus by transgenesis and biolistic transfection. Methods Enzymol *393*, 579-592.

Maravall, M., Mainen, Z.F., Sabatini, B.L., and Svoboda, K. (2000). Estimating intracellular calcium concentrations and buffering without wavelength ratioing. Biophys J *78*, 2655-2667.

Tsien, R.Y. (1989). Fluorescent probes of cell signaling. Annu Rev Neurosci *12*, 227-253.
