# Supplementary figures and images for: A Gq-Ca2+ Axis Controls Circuit-Level Encoding of Circadian Time in the Suprachiasmatic Nucleus
Source: Neuron. 2013 May 22;78(4):714–28. doi: 10.1016/j.neuron.2013.03.011 (PMC3666084; doi:10.1016/j.neuron.2013.03.011)

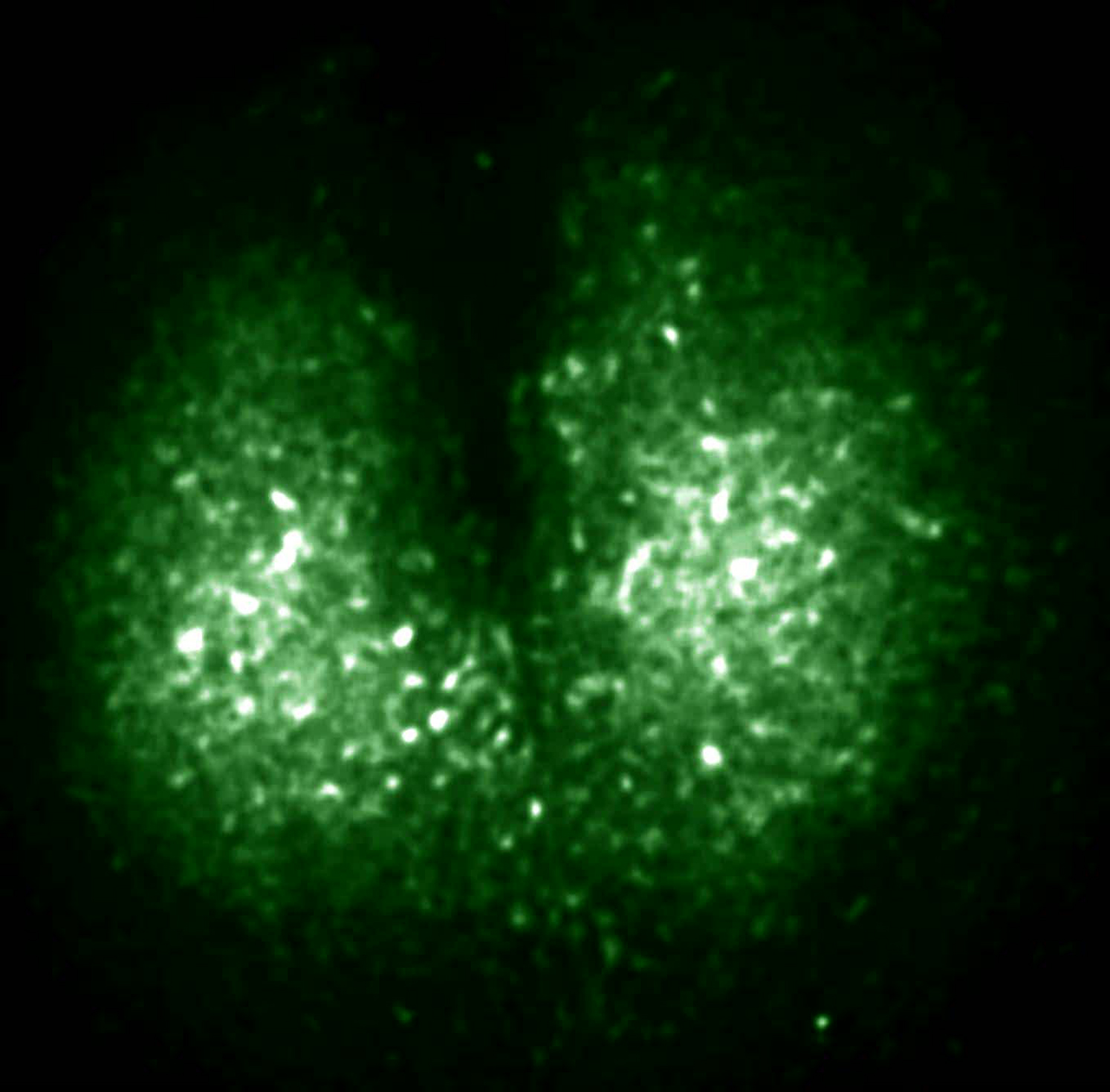

Supplement: Movie S2. Representative Time-Lapse Video Recording of a Wild-Type SCN Organotypic Slice Transduced by the AAV:Syn-GCaMP3 — LUT code: thallium. See also Figure 4. [file mmc3.jpg]

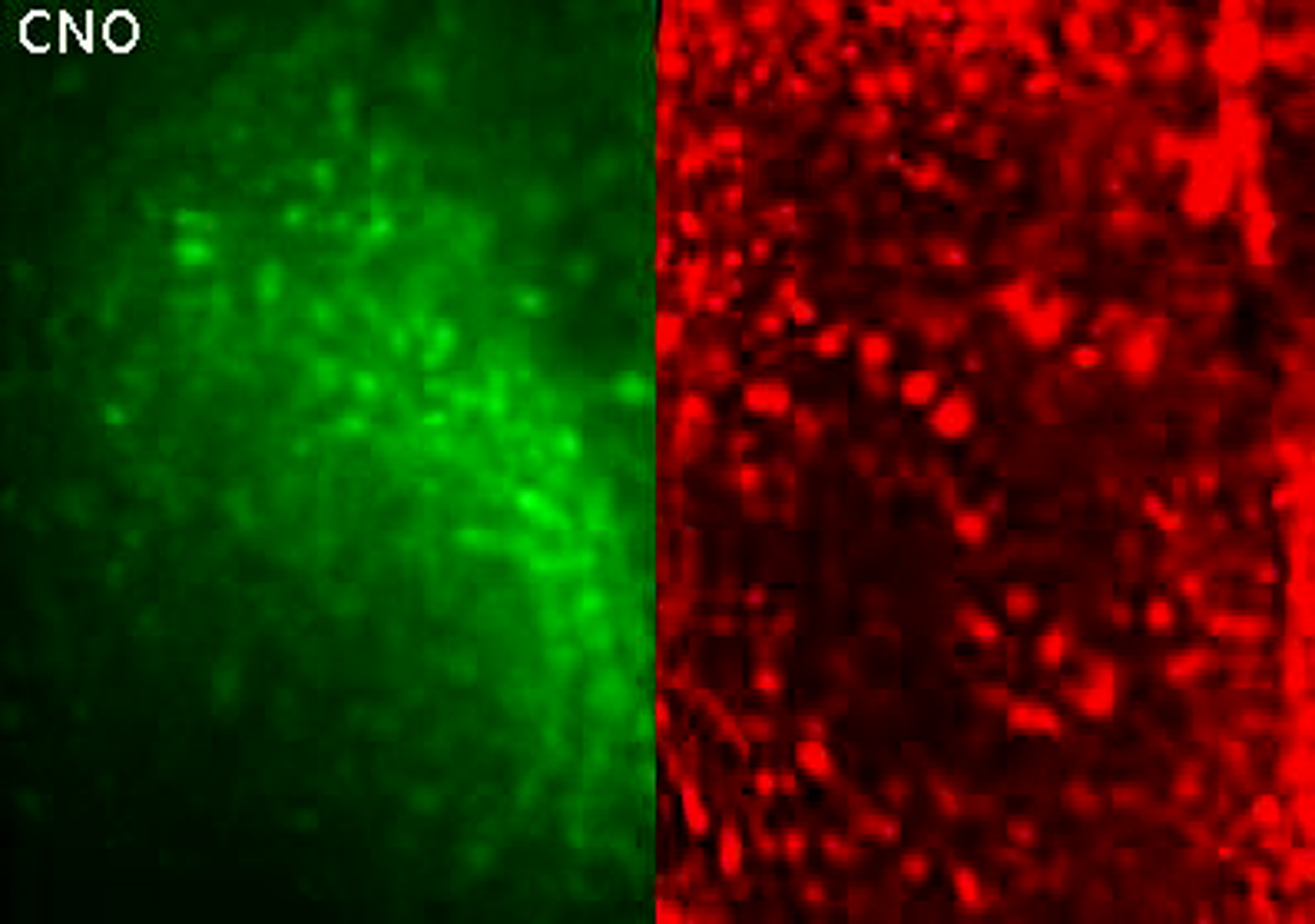

Supplement: Movie S4. Representative Time-Lapse Video Recording of a Pharmacogenetic Gq Activation Experiment — Wild-type SCN slice transduced by AAV:Syn-GCaMP3 and LV:hM3DGq-IRESmCherry was recorded before CNO addition, in the presence of CNO and after washing out. CNO addition is marked on the movie. LUT code: Syn-GCaMP3 = green; LV:hM3DGq-IRESmCherry = red. See also Figure 5. [file mmc5.jpg]

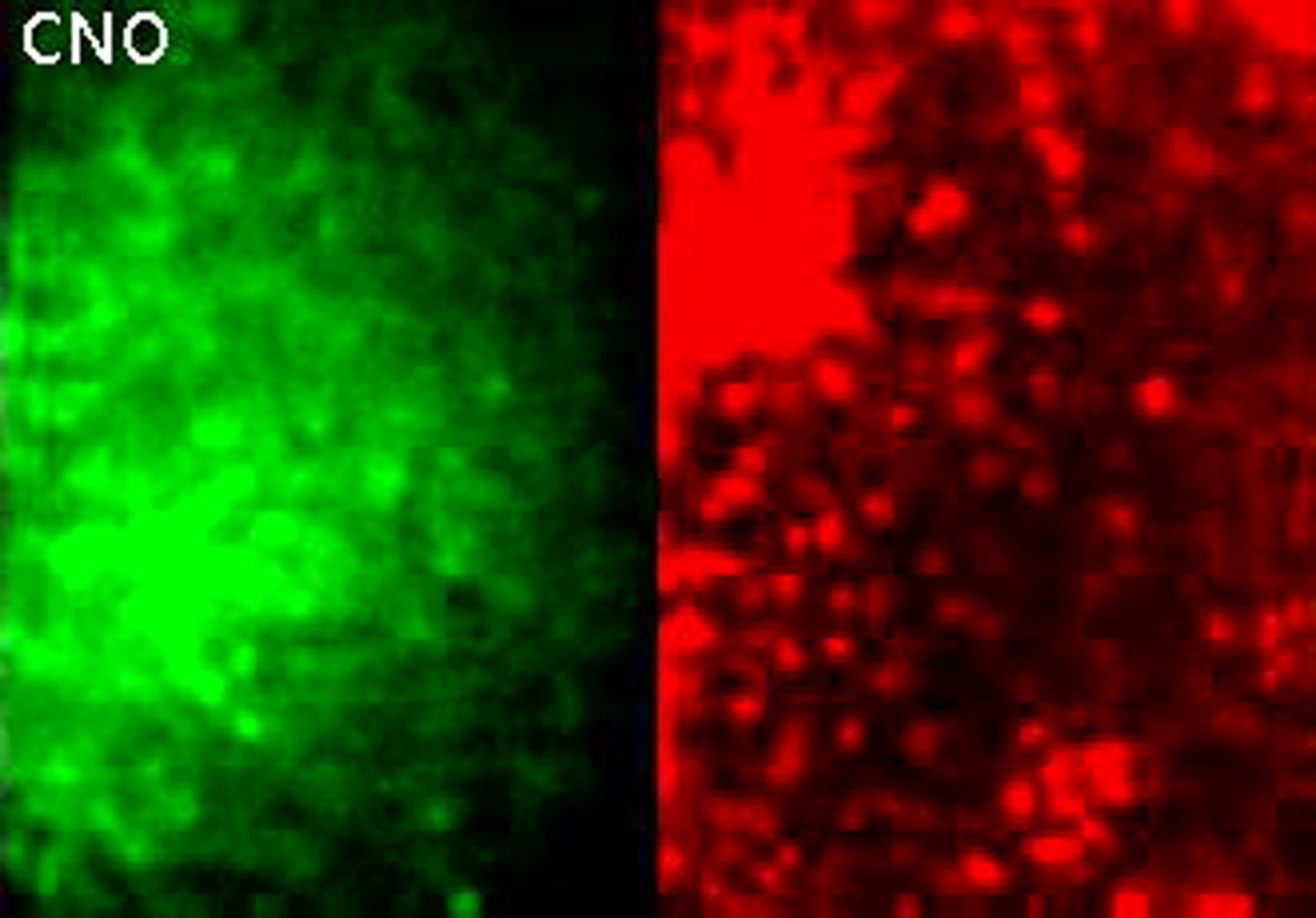

Supplement: Movie S5. Representative Video Time-Lapse Recording of a Pharmacogenetic Gs Activation Experiment — Wild-type SCN slice transduced by AAV:Syn-GCaMP3 and LV:Syn-rM3/β1Gs-IRESmCherry was recorded before CNO addition, in the presence of CNO and after washing out. CNO addition is marked on the movie. LUT code: Syn-GCaMP3 = green; LV:Syn-rM3/β1Gs-IRESmCherry = red. See also Figure 5. [file mmc6.jpg]

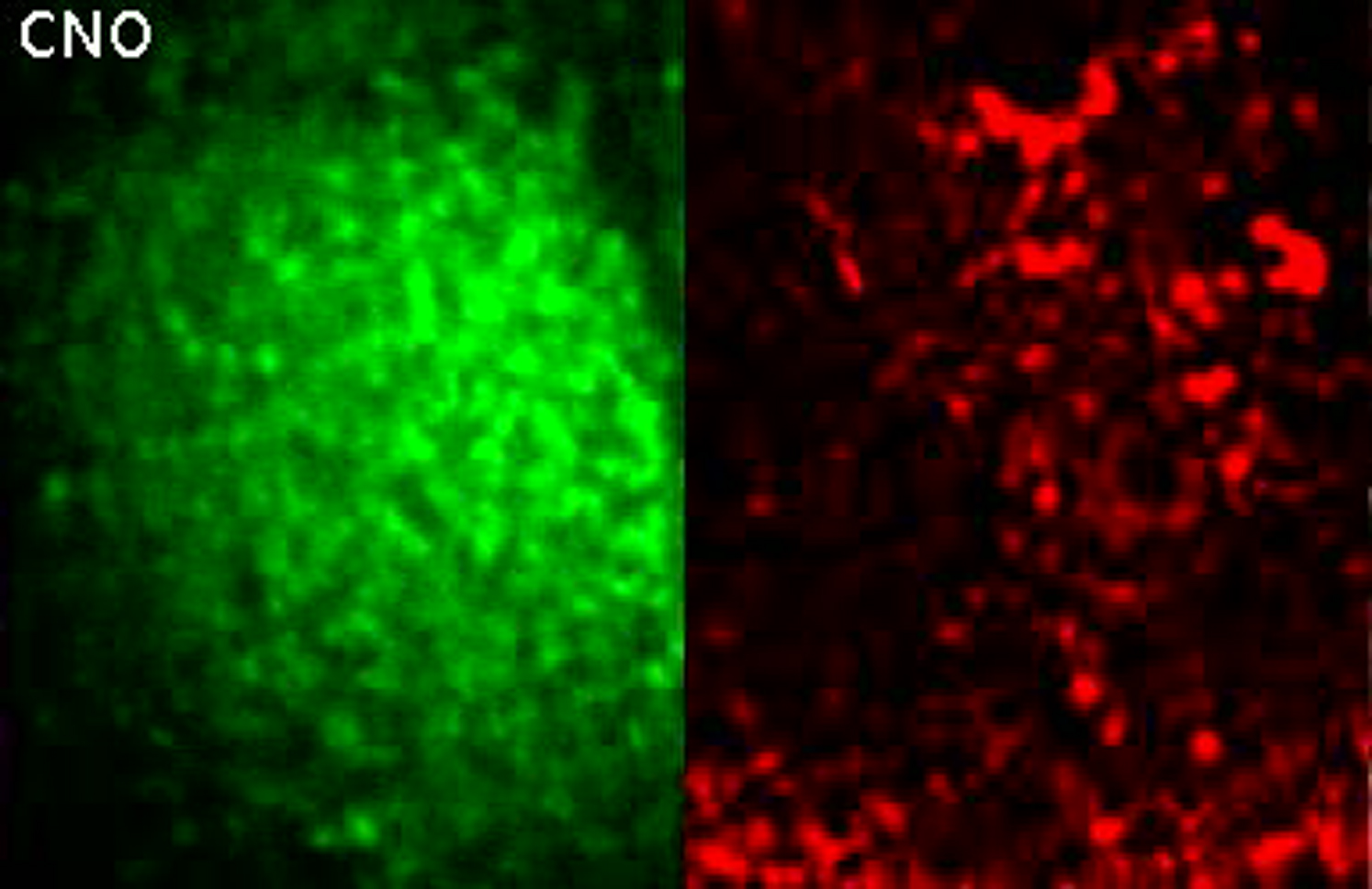

Supplement: Movie S6. Representative Time-Lapse Video Recording of a Pharmacogenetic Gi Activation Experiment — AAV:Syn-GCaMP3 and LV:hM4DGi-IRESmCherry was recorded before CNO addition, in the presence of CNO and after washing out. CNO presence is marked on the movie. LUT code: Syn-GCaMP3 = green; LV:hM4DGi-IRESmCherry = red. See also Figure 5. [file mmc7.jpg]
